# 3D photogrammetry as a tool for studying erosive processes at a Roman coastal site: the case of the Roman fish-salting plant at Sobreira (Vigo, Spain)

**DOI:** 10.1007/s12520-022-01508-3

**Published:** 2022-01-27

**Authors:** Adolfo Fernández Fernández, Patricia Valle Abad, Alba Antía Rodríguez Nóvoa

**Affiliations:** Faculty of History, Department of Art, Geography and History, GEAAT, University of Vigo, Olga Gallego 1st floor, 28, 32004 Ourense, Spain

**Keywords:** Marine erosion, Roman industry, Photogrammetric models, Virtual archaeology, CloudCompare, Project GaltFish

## Abstract

Rising sea levels, along with other biological and human factors, have increased erosion rates at a number of important sites located along the Atlantic coastline. Project GaltFish implemented a series of contingency measures to record some of these sites before they degraded further or totally disappeared. This process involved detailed photogrammetric recording of some of the sites under threat over a set period of time. One of the sites selected for this project was Sobreira (Vigo, Galicia): a Roman fish-salting factory which was partially destroyed by building activity in the 1980s and the remains of which are under threat from marine erosion and human action. In order to study the site, two photogrammetric models were created to examine the effect of erosive processes across the course of one year. The results illustrate that photogrammetry is an efficient tool for recording and analysing the issue of erosion. The data compiled helped in designing additional action in the factory, which was subject to a rescue excavation to record and help protect the site from further damage. This paper presents the results of this project, as well as the methodology used to produce the models, the data generated and their analysis. It is argued that the methodology can be used to collect and analyse data from other sites, and that this data could inform the political/administrative decision-making processes which concern the future management and preservation of archaeological sites under threat.

## Introduction: background and setting

The presence of a large number of factories for the processing of marine resources on the Atlantic coastline of ancient *Gallaecia* led, in 2016, to the launching of the research project GaltFish—Salt and Fish Salting in Ancient *Gallaecia*: looking for the origins of the Galician canned fish industry. The project aimed to collect information about Roman fish-salting factories in the region in order to better understand the operation of these establishments and the commercialisation of their products by analysing commercial routes and links between different regions of the empire (Fernández Fernández and Morais 2017; Fernández Fernández 2017a).

Previous works and publications provided merely lists of long known fish-salting factories, most of them without recent scientific excavations. Besides, some of them had a dubious adscription, due to the scarce and barely consistent data provided about them. However, after a data collection and a singular analysis of each case, it was possible to individualise at least 15 “certain” factories in the coastal area located between the mouth of the Miño River and the city of Gijón,^1^ discarding some of the sites that were traditionally considered fish-salting factories.^2^ Adding to this group two more factories, located between the Miño and the Douro River^3^; as well as a group of Roman coastal fish factories with unknown functionality, among which the site of Sobreira was included, considered before its excavation as a *villa a mare* (Hidalgo Cuñarro and Viñas Cuñe 1999: 88–89). Most of these factories, as well as other Roman coastal sites, have been listed in different works (Lomba Portela 1987; Naveiro López 1991), some of them concerning new researches, like the one undertaken in Plaza del Marqués in Gijón (Fernández Ochoa 1994; Fernández Ochoa and Martínez Maganto 1994) and more recent ones related to rescue excavations, like the factories discovered in Vigo’s inner city, denoting the importance of the Rias Baixas as the major fish-salting production area of the northwest of the Iberian Peninsula (Suárez Piñeiro 2003; Currás Refojos 2007). Unfortunately, very few sites have monographic publications, except for the aforementioned Gijón (Fernández Ochoa 1994) and others like Fiunchal (Castro Carrera 1992), Bueu (Díaz García 2015), Marqués de Valladares (Torres Bravo et al. 2007) or the recent case of Igrexiña (Gorgoso López and Acuña Piñeiro 2016).

GaltFish Project included archaeological works on various fish-salting factories (Sobreira-Vigo, Adro Vello-O Grove and O Naso-Illa de Arousa). The aim of this was to verify if some of these dubious sites, like Sobreira and O Naso, were, in reality, fish-salting factories, while in other cases, like Adro Vello, the objective was to collect new data about the chronology of the site, as well as to obtain a detailed and complete plan of the factory. Besides, during this intervention, an important ensemble of ichthyofauna was recovered inside one of the vats.

**Fig. 1:**
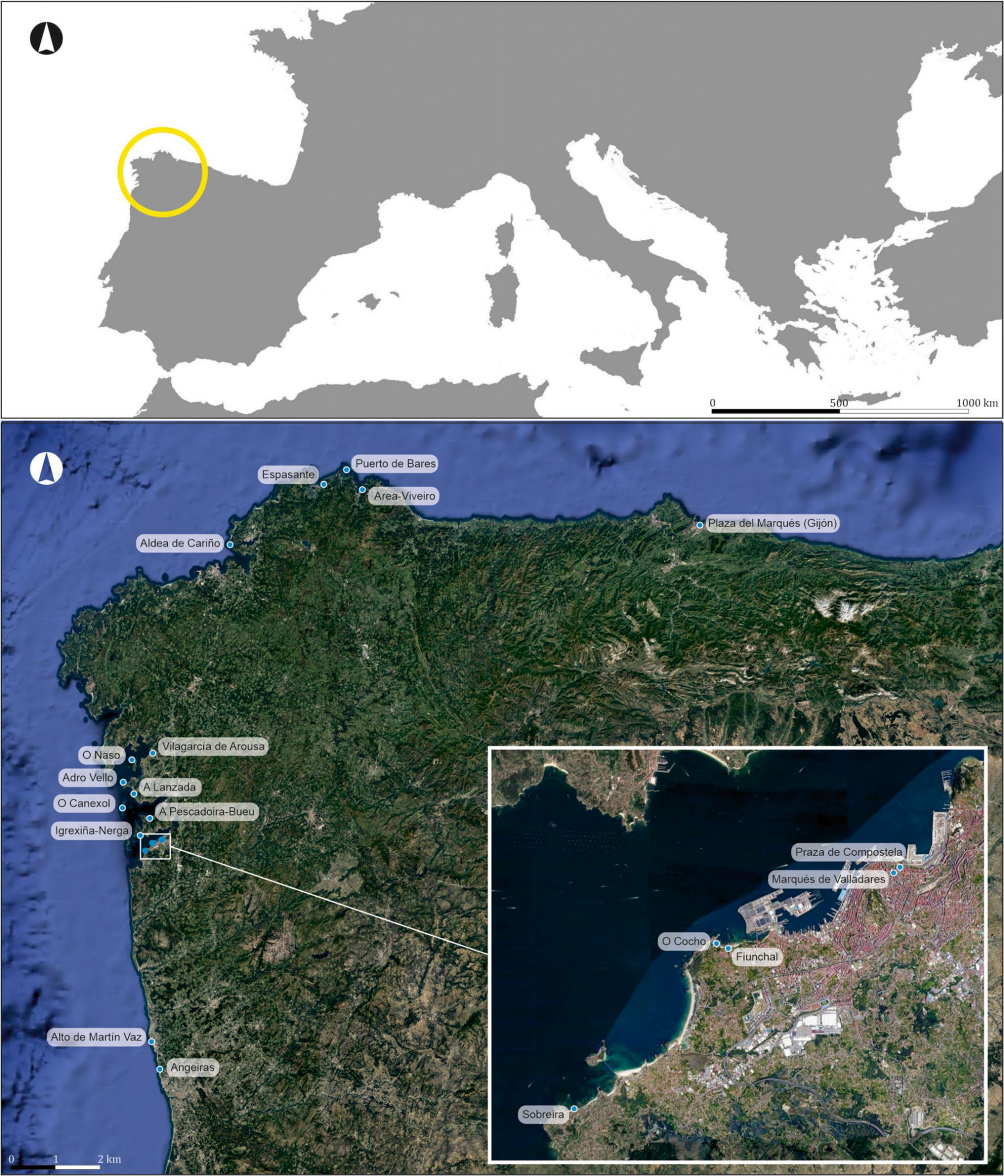
Map showing the fish-salting factories located between the mouth of the Douro River and the city of Gijón

These works provided first-hand evidence of the organisation and operation of these factories and resulted in the publication, in the database RAMPPA (http://ramppa.uca.es/), of 19 Roman fish-salting factories, located between the mouth of the Douro River (Portugal) and the Basque–French border (Fig. 1). These factories included the sites of O Naso (Illa de Arousa) and Sobreira (Oia, Vigo), which was proven to be a Roman fish factory (Fernández Fernández 2017c) rather than a *villa a mare*, as previously thought (Hidalgo Cuñarro and Viñas Cue 1999).

Besides, GaltFish improved the knowledge of other sites related to the exploitation of the marine resources during the Roman period, as solar evaporation salt pans, particularly the one of O Areal (Castro Carrera et al. 2019; Iglesias Darriba et al. 2017). But also, the analysis of its trade, from the study of the amphorae production of Bueu, from where a large amount of the salt-fish products of the Rias Baixas were exported (Fernández Fernández 2016a, b, 2017a; Fernández Fernández and Morais 2017). It was proven that barrels were also used for the transport of products, probably for the North European markets, while the amphorae were traded in the Lusitanian and Mediterranean markets, as demonstrated by the presence of San Martiño de Bueu 2 amphorae in Oporto, Lisbon, Faro, Seville, the Balearic Islands and Rome (Fernández Fernández 2017a).

Although addressing preservation issues was not among GaltFish’s primary aims, the project also addressed these. The sites under study owing to their coastal location are affected by erosive processes, which are largely caused by sea dynamics. In addition, many of them are located in densely inhabited areas, resulting in further preservation threats. A paradigmatic example of this in Galicia is Guidoiro Areoso, an islet in Ría de Arousa where a series of important megalithic archaeological remains have suffered in recent years the effect of increasingly violent tides and storms (Blanco-Chao et al. 2015; Rey-García and Vilaseco Vázquez 2012).

In recent years, a number of projects have been launched to implement mitigating measures to slow or halt the deterioration of archaeological sites affected by climate change and other environmental issues, such as project ALeRT (https://alert-archeo.org/), SCAPE/Sch@arp (http://www.scharp.co.uk/), Rapid Coastal Zone Assessment (https://archaeologydataservice.ac.uk/archives/view/rczas/), Arfordir (http://www.ggat.org.uk/arfordir/) or STORM (http://www.storm-project.eu/), focused on prevention and mitigation as well as the design of specific planning policies. Project GaltFish, on a more local level, aims to measure the effects of these erosive processes—both natural and human—on coastal archaeological sites in order to propose specific measures for the preservation of these sites at the local/regional level.

**Fig. 2:**
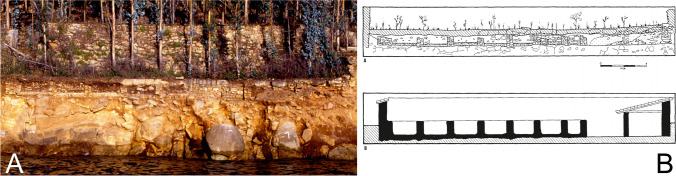
**A** Remains of the fish-salting factory of Cariño (A Coruña). **B** Reconstruction of the remains on the cliff (Naveiro López 1991:103, Fig. 23)

**Fig. 3:**
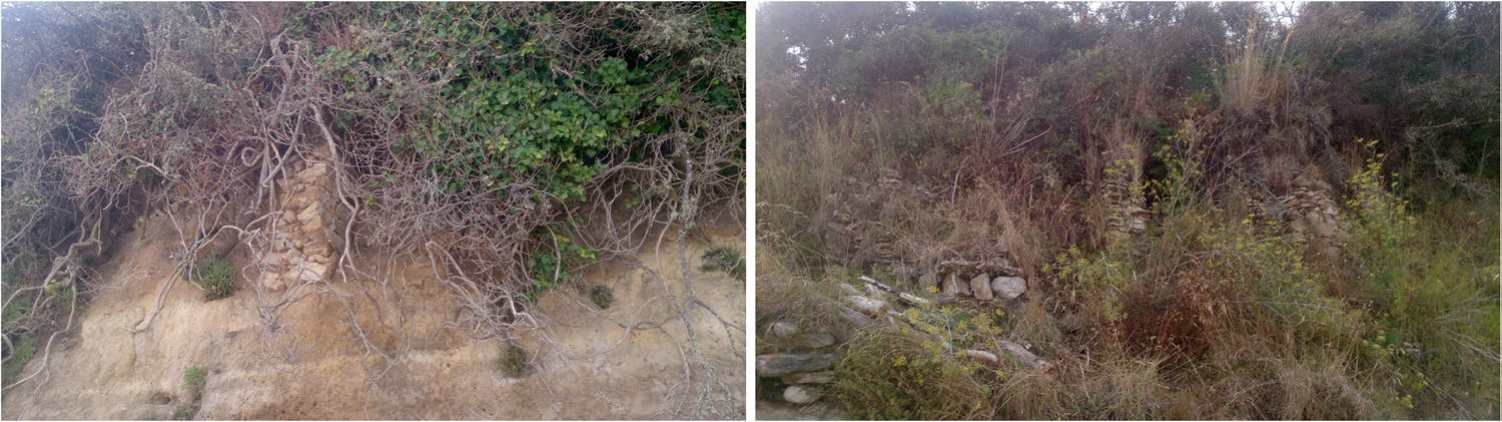
Wall remains belonging to a fish-salting factory on the sandy coastline of O Canexol (Illa de Ons, Galicia)

**Fig. 4:**
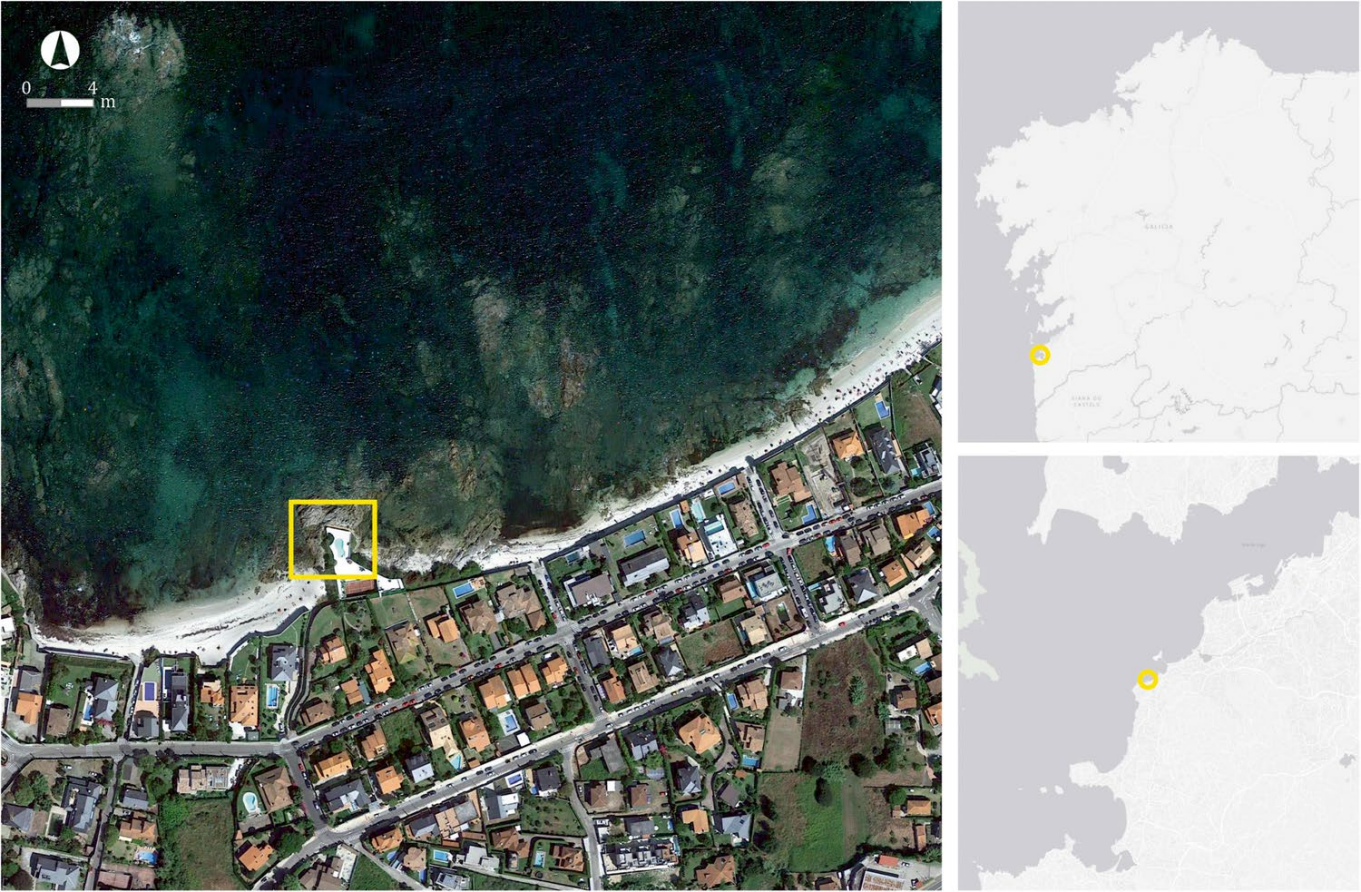
Location of the site of Sobreira. Maps: Google Satellite and ESRI

**Fig. 5:**
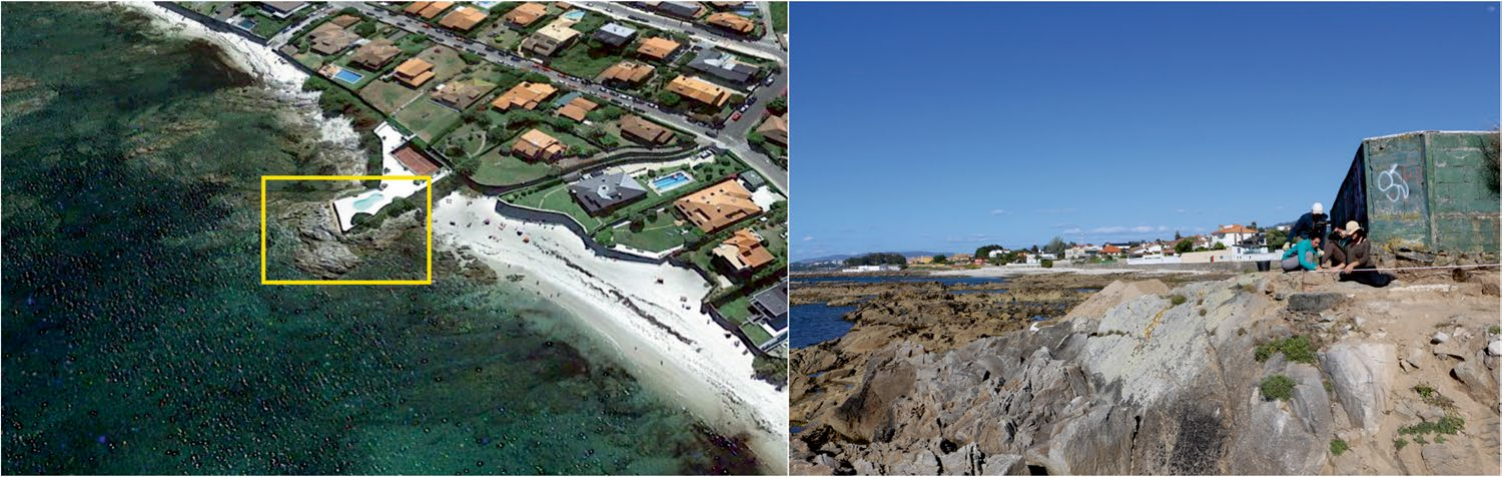
Left: aerial view of the site of Sobreira (Google Earth). Right: photo taken during the archaeological works, where it is possible to see the relation between the site and the sea

Concerning some of our Roman fish-salting factories, the problem is severe; some are close to disappearing completely. The most extreme cases are located on Galicia’s northern coast: for instance, the factory of Cariño in A Coruña (Fig. 2) and the beach site of Area in Lugo. The Rías Baixas are also affected by this problem, the worst cases being the sites of Canexol (Fig. 3), on the island of Ons, and Sobreira, in Vigo. Owing to its location, Sobreira was deemed to be the most suitable site for carrying out a pilot project to monitor the erosive processes, both natural and human, that threaten the archaeological remains. The fish-salting factory of Sobreira is located on a rocky outcrop known as Punta Sobreira, between the beaches of Sobreira and Fuciños, in the municipality of Vigo (Figs. 4 and 5). The site was discovered in the 1950s and was greatly damaged by the construction of a private sports facility in the 1980s. Damage was extensive, and the only features to survive are those located in the northernmost sector of the promontory (which is the most exposed to sea dynamics), the area to the west of the sports facility, and probably also beneath it.

**Fig. 6:**
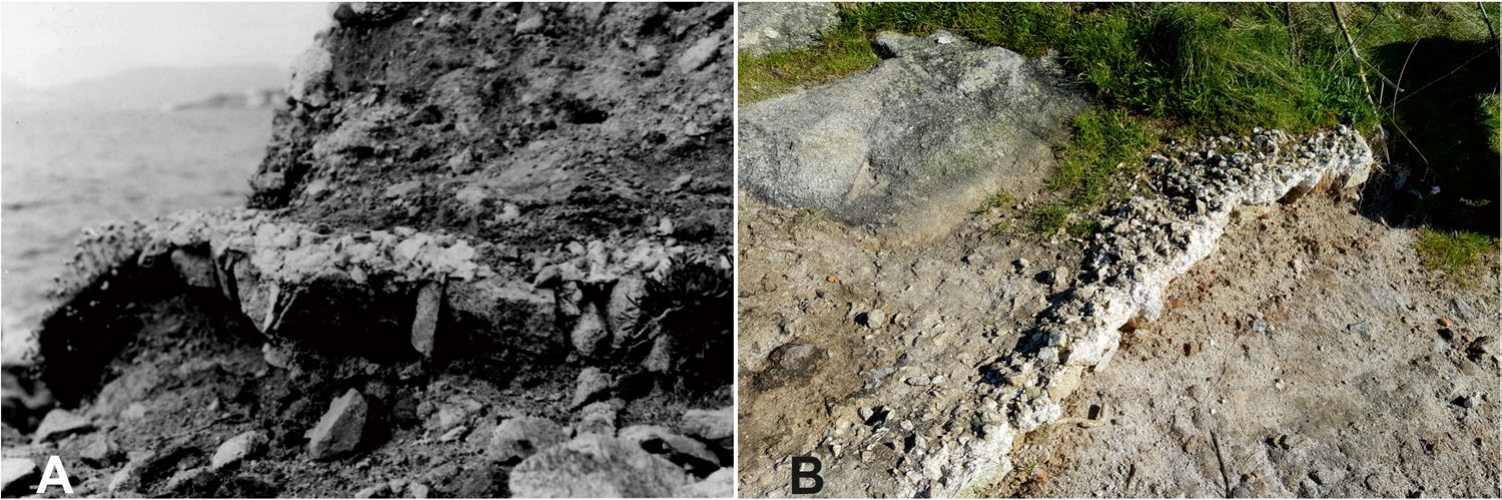
**A ** Mortar remains covered by a thick layer of soil in 1984 (Photo: Museo Municipal Quiñones de León, Vigo). **B** The same mortar feature in May 2016

Several photographs taken by Hidalgo Cuñarro and Costas Goberna on 2nd May 1984 show the remains of a quartz mortar pavement sitting on a layer of granite blocks laid out diagonally; these features were covered by a layer of soil, several centimetres thick (Fig. 6A). The photographs also illustrate a large wall built with irregular masonry blocks on the easternmost limit of the beach, which has been interpreted as the eastern wall of the Roman building. In 1999, Sobreira was included in a paper about new archaeological sites in Vigo, considered a *villae*, similar to the nearly Toralla and the one of Riós (Hidalgo Cuñarro and Viñas Cue 1999: 88–89). This paper classified also as *villae* the sites of Fiunchal and O Cocho, later catalogued as fish-salting factories (Fernández Fernández 2017b, 2017d). This last site, O Cocho, located in a beach with the same name, after its detailed analysis during the development of the project, was considered a factory, therefore being included in the catalogue of the RAMPPA (Fernandez Fernández 2017d).

## Case study: marine and human erosion at the site of Sobreira

The first visit to the site in 2016 revealed that the conditions of the site had changed considerably since its discovery, to the point that the northern sector was in danger of imminent destruction. The area located between the swimming pool and the sea had been used as a pathway, and this had severely eroded the surface. The mortar pavement, which in 1984 was still under a layer of soil (probably the mortar lining of a salting cistern), was being used as a step by beachgoers to travel from one beach to the other (Fig. 6B).

In addition to the mortar, which was visible and totally exposed, the ends of at least two walls were visible elsewhere on the site. These walls were also traversed by the pathway that ran across the promontory. In addition to human action, aggressive sea erosion had led to the total loss of the northern face of the site. During the winter months, the surf and high tides reach the wall of the chalet built over the site (sometimes even going far beyond this point), and the effects of the resulting erosion have left the wall foundations exposed. As a result, the northernmost features were particularly exposed. The Roman wall identified to the west, in contrast, was covered by dense vegetation, which protected it from erosion. Moreover, since it was far away from the pathway used by beachgoers, it remained unaffected by human action.

In addition to this, the presence of a number of increasingly large sinkholes threatened the integrity of the few layers of soil that still protected the remains, as well as that of the surviving Roman walls. Furthermore, the uncontrolled proliferation of undergrowth, both along the walls of the chalet and the site’s frontal facade, exacerbated the loss of archaeological levels.

Under these conditions, the total disappearance of the site seemed imminent. This made the site ideal for monitoring the effects of erosion on coastal sites under severe threat, over the course of a year. The results highlighted the need for certain mitigating measures: the first priority was to record as much archaeological evidence as possible and, as far as practicable, to preserve the original structure of the factory, considering the level of destruction already sustained by the site and the impossibility of removing the pathway that runs across the promontory.

## Monitoring change: recording methodology

**Fig. 7:**
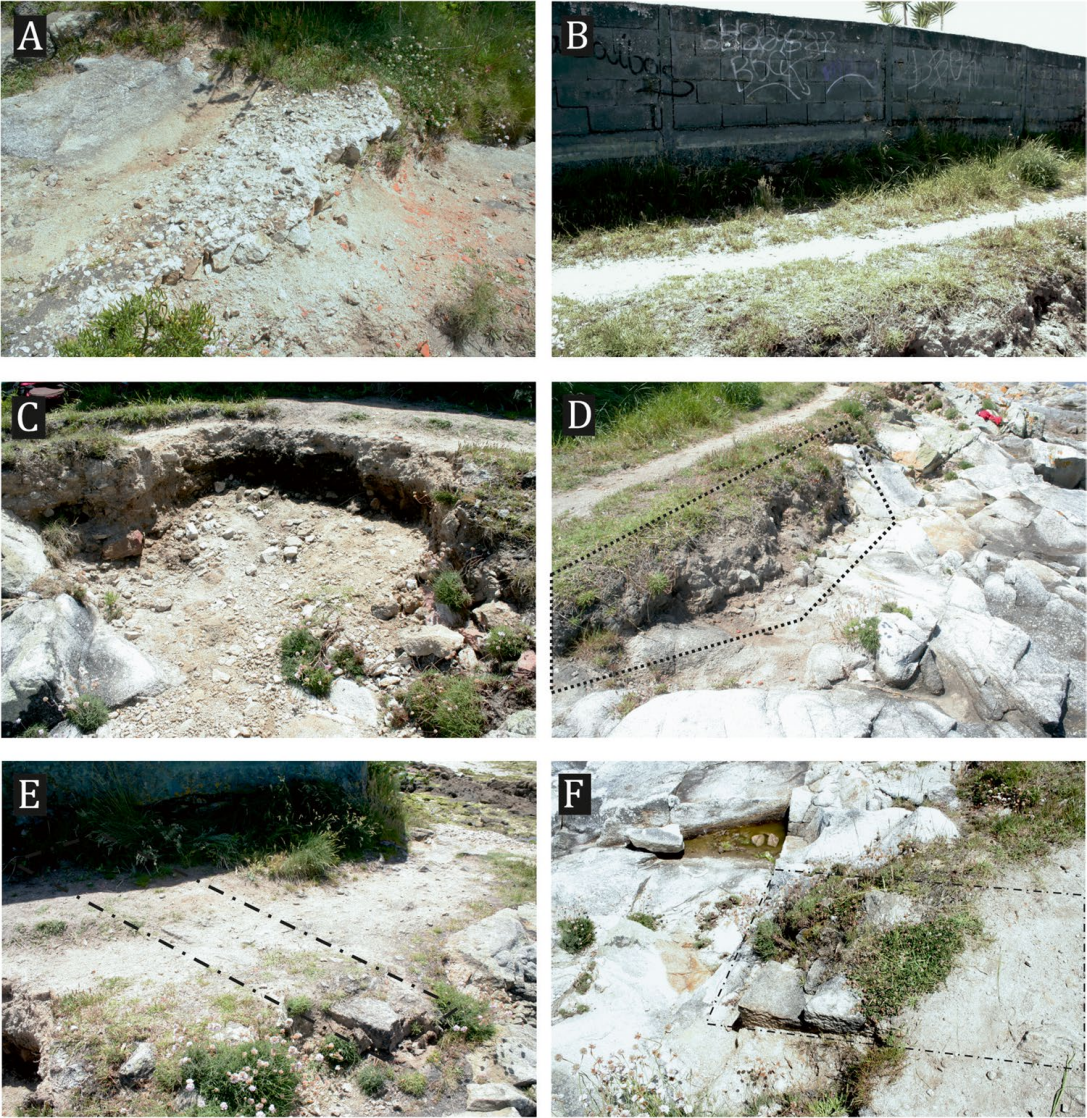
Location of the affected areas, whose degradation was noticeable during the visit to the site in 2016. (**A**) Partially destroyed mortar; (**B**) presence of weeds; (**C–D**) loose of material in the front side of the path; (**E–F**) visible and exposed walls

Although some of the erosive processes affecting the site were plainly visible (Fig. 7), it was deemed necessary to monitor them, allowing us to detect less obvious processes. We were thus able to determine the exact nature of the threats and the associated erosion rates. The methodology followed consisted of taking one photogrammetric model of the site and another 1 year later, as this would theoretically allow us to measure the effects of erosive processes upon the site accurately.

In essence, the research protocol comprised three stages: taking the photographs, generating the 3D models and comparing both models. Efforts were made to ensure that the conditions prevailing when both sets of photographs taken (in May 2016 and May 2017) were as similar as possible in order to generate as closely comparable models as practicable, thus minimising the margin of error. Considering the magnitude of the erosive processes observed in the different visits to the site, the final survey should be accurate enough to quantify centimetre differences between both shots.

### Taking the photographs

The initial sequence of photographs was taken in late May 2016 (Sequence 1 – S1), and the second around the same dates in May 2017 (Sequence 2 – S2). We attempted to match as closely as possible the weather and light conditions for both sessions, in order to avoid differences that could have a negative effect on the processing and comparison of both sequences. Some changes, however, had to be made: for instance, the second session (S2) had to be undertaken in the afternoon rather than midday to match the low-tide conditions prevailing during the first session (S1). This leads, however, to a notable light-shadow difference between both models, mainly visible in the final textures.

The photographs were taken without the aid of a tripod or any other physical support, owing to the rugged terrain conditions. In addition, the two sequences were taken with different cameras, which slightly affected the quality of the photographs and consequently the final models (Table 1).

**Table 1:** Technical specifications of the two photographic sequences, including camera configuration, the number of photographs and the results of image processing carried out with the aid of Agisoft Metashape®

	*Sequence 1*	*Sequence 2*
*Date*	May 2016	May 2017
*Climatic conditions*	Sunny	Sunny
*Time frame*	Midday (12.00–14.00)	Afternoon (18.00–20.00)
*Camera*	EOS 350D Digital (18 mm)	EOS 700D (18 mm)
*Camera settings*	Automatic exposure and shutter speed	Automatic exposure and shutter speed
*Focal length*	18 mm	18 mm
*Photographs*	538	415
*Coverage area*	167 m^2^	163 m^2^
*Tie points*	528,079 (medium)	789,000 (medium)
*Dense cloud*	15,832,000 (medium)	24,130,000 (medium)
*Mesh*	3,157,000 (medium)	4,841,000 (medium)
*Decimated mesh*	3,000,000	3,000,000
*Texture*	Mosaic (16,096 × 1)	Mosaic (16,096 × 1)
*Ground resolution*	1.1 mm/pix	0.869 mm/pix
*Reprojection error*	0.809 pix	0.748 pix

**Fig. 8:**
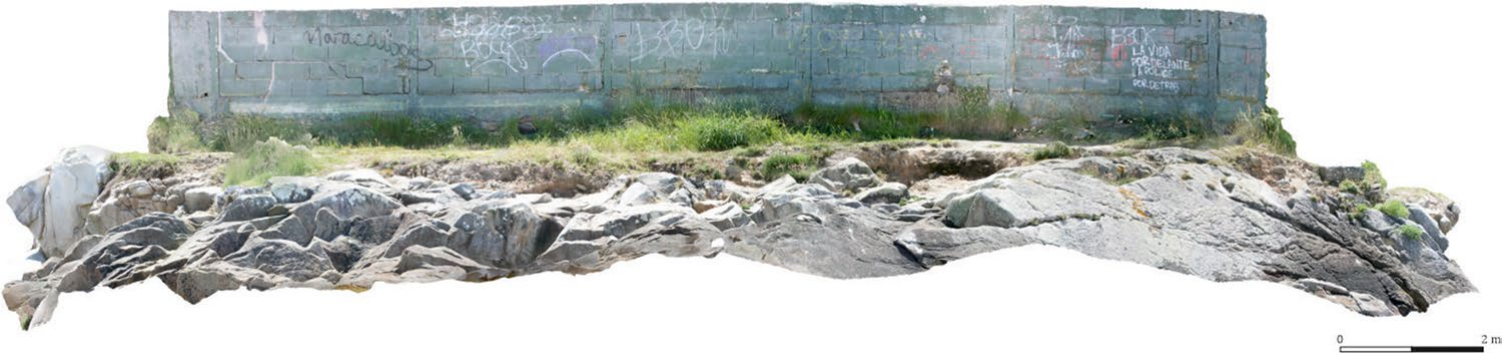
Frontal view, from the north, of the first 3D model for Sobreira, which clearly illustrates the rugged nature of the terrain

Both sequences followed a linear pattern. Photographs were taken along a line with a separation interval of approximately 1 m, and a minimum overlap of 60–70%. Photographs were taken from east to west and from west to east along the pathway that crosses the promontory, resulting in a full photographic sweep of the site. Areas which were deemed to be blind spots, especially several sectors of the rocky outcrop, were photographed separately. In order to ensure that the whole site was covered, additional photographs were taken from farther away, resulting in general overviews that facilitated the assembling of different photographs to generate the final model (Fig. 8).

**Fig. 9:**
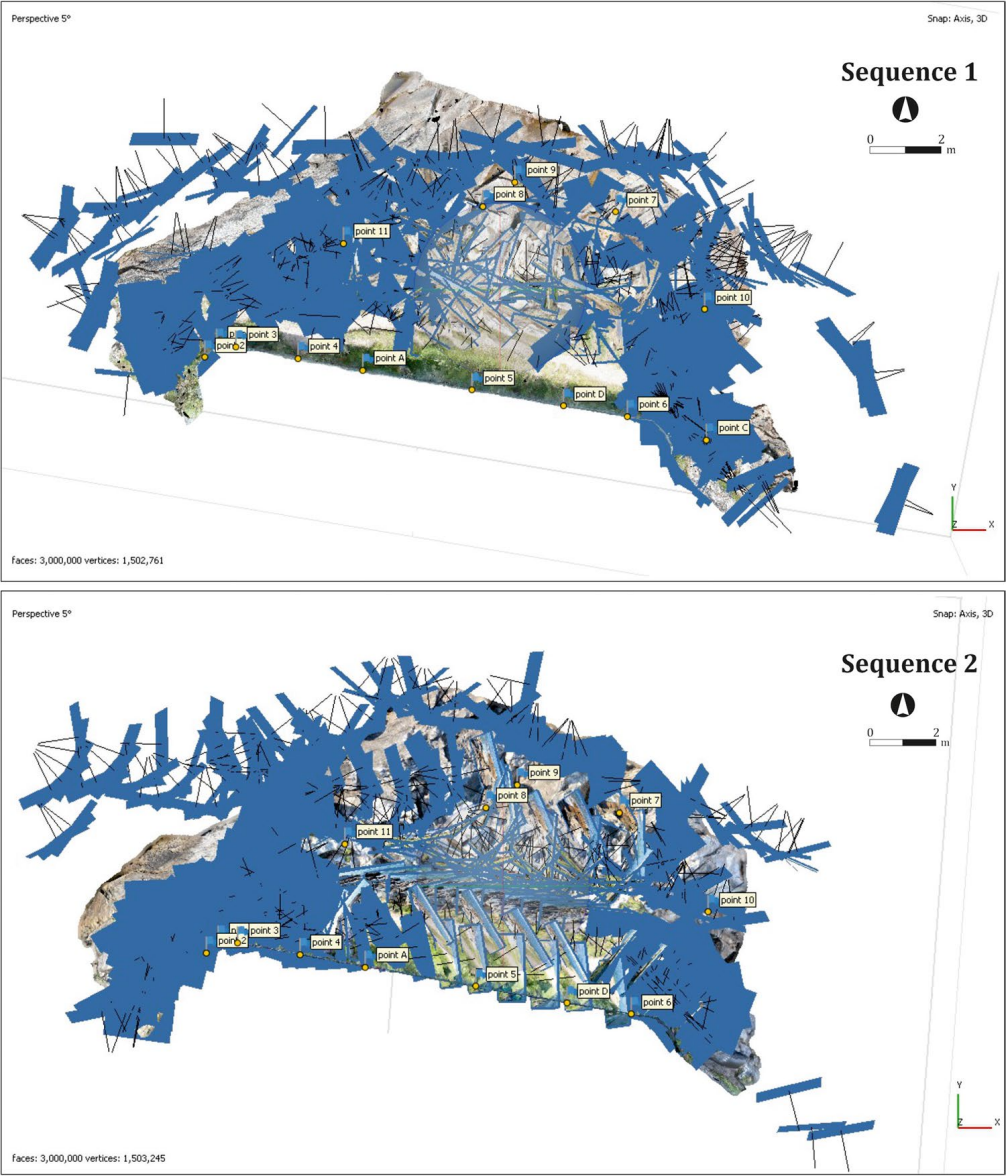
Screenshots from Argisoft Metashape © showing the spatial orientation of the imagery for 2016 (above) and 2017 (below)

A previous evaluation of the terrain revealed that, even using a linear pattern, some of the photographs could not be taken from a regular distance—for example, those taken from the rocky outcrop—as the terrain presented height differences of approximately 1 m and there were few stable standpoints. Besides, some of the blind spots were not fully accessible, changing the distance and the perspective from where they had to be registered (Fig. 9). The first shot (S1) was done during midday, while the sun projects more light/shadow differences in the uneven terrain. Besides, the time used to evaluate the area before its registry showed quick light changes on the terrain surface—covered by water in several spots and with some shadowed blind spots—that would affect the final product. Thus, considering the time needed to take the photographs, it was decided to set the exposure and shutter speed of the camera in automatic mode, to minimise the contrast among the different shots, to assure an acceptable image capture, despite knowing that it could produce minor variances in the final mesh. Aside from this, the focal length of the camera was always established in 18 mm, repeating these settings for the sequence of 2017.

### Generation of the model

**Fig. 10:**
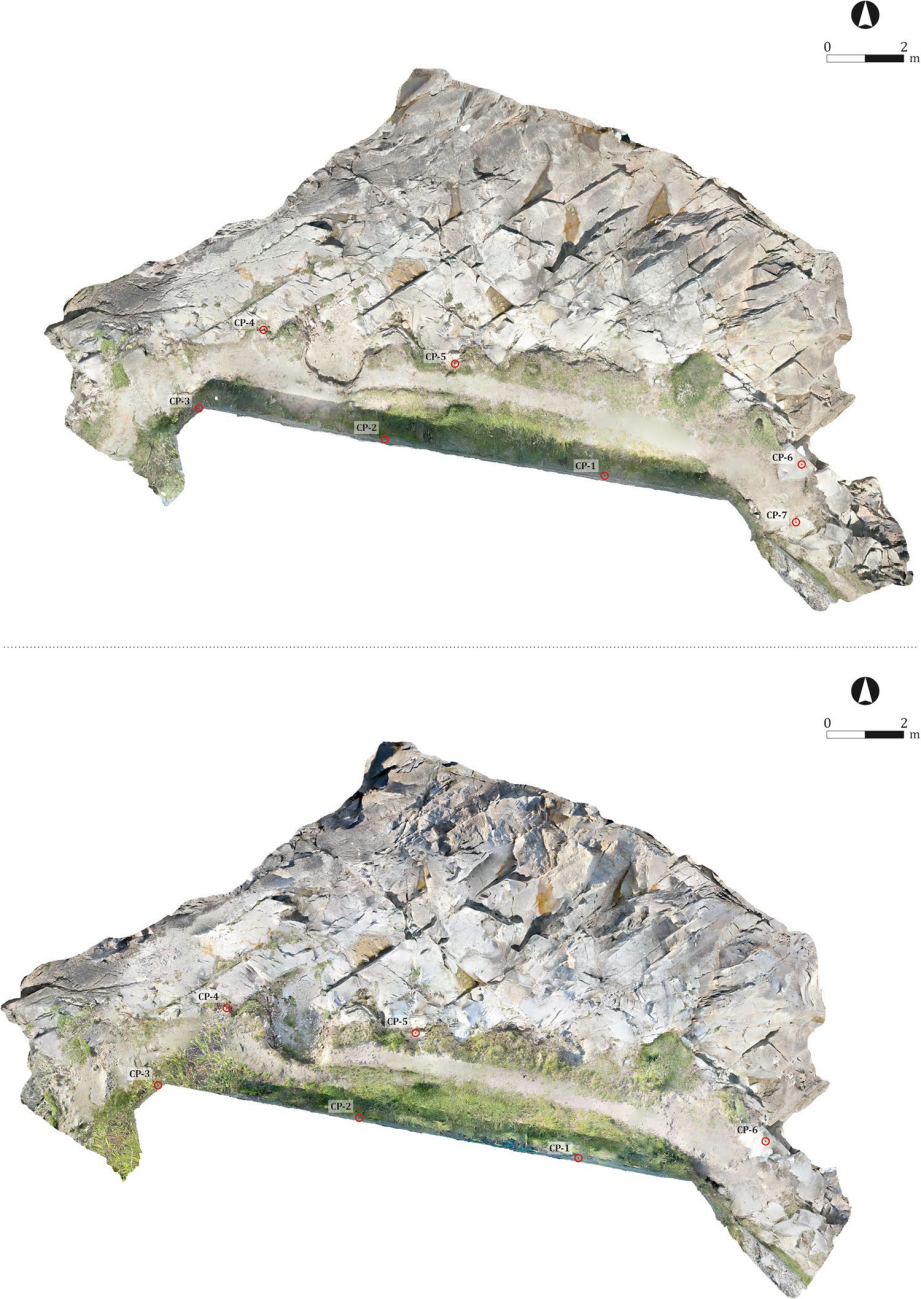
Photogrammetric models of the site of Sobreira with the distribution of the GCPs. Above: 3D model reali*s*ed in 2016 of the site of Sobreira; below: 3D model reali*s*ed in 2017

Image processing was carried out with the aid of Agisoft Metashape® software, version 1.6.5. All processes were undertaken at medium resolution (Table 1), as it was enough to create detailed and still manageable models. As illustrated by the figure (Fig. 10), some sectors—characterised by the presence of dense vegetation or flooded areas—could not be adequately modelled, leading to small errors in the final mesh. These inadequacies, however, did not hamper the comparison of the models.

The results, as presented in Table 1, show that the model resulting from Sequence 2 was more accurate than that yielded by Sequence 1, whose final model surface was noticeably less detailed. Therefore, to ensure an adequate post-processing, it was decided to decimate both meshes to 3,000,000 faces, which to some extent brought the models closer together, lightening the meshes and easing the post-processing works.

In both cases, the final texture applied the highest resolution settings that the equipment in use could handle (Fig. 8), to ensure a high visual quality for both models. Similarly, the models were oriented through three ground reference points—located in specifical spots of the rocky outcrop and the visible structures of the factory—that worked as GCPs (Table 2). The terrestrial reference system used was ETRS89/UTM zone 29 N (EPSG: 25,829).

**Table 2:** RMSE for the GCPs and check points of both sequences

	*Sequence 1*	*Sequence 2*
	*GCPs*	*GCPs*
*Count*	7	6
*X error (mm)*	2.80002	0.83085
*Y error (mm)*	0.37322	0.21059
*Z error (mm)*	0.93592	0.35436
*XY error (mm)*	2.82478	0.83112
*Total (mm)*	2.97579	0.90351

### Compared model

The final step was carried out with the aid of CloudCompare® open-access 3D processing software, version 2.11.3. This aimed to obtain, measure and quantify hard evidence for something that was already visible to the naked eye: namely, the progressive destruction of the site. However, before carrying out the comparison, both models, in OBJ format, were cut in Blender® (v. 2.8), to obtain a similar surface and avoid possible errors produced by meshes with different shapes.

Imported both meshes in CloudCompare®, the comparison consisted on a process known as “Compute Cloud/Mesh distance”. Firstly, Sequence 1 was used as a reference model, while Sequence 2 was used as a comparison model. This means that data concerning the loss or gain of material on the ground were presented over the model based on Sequence 2.

**Fig. 11:**
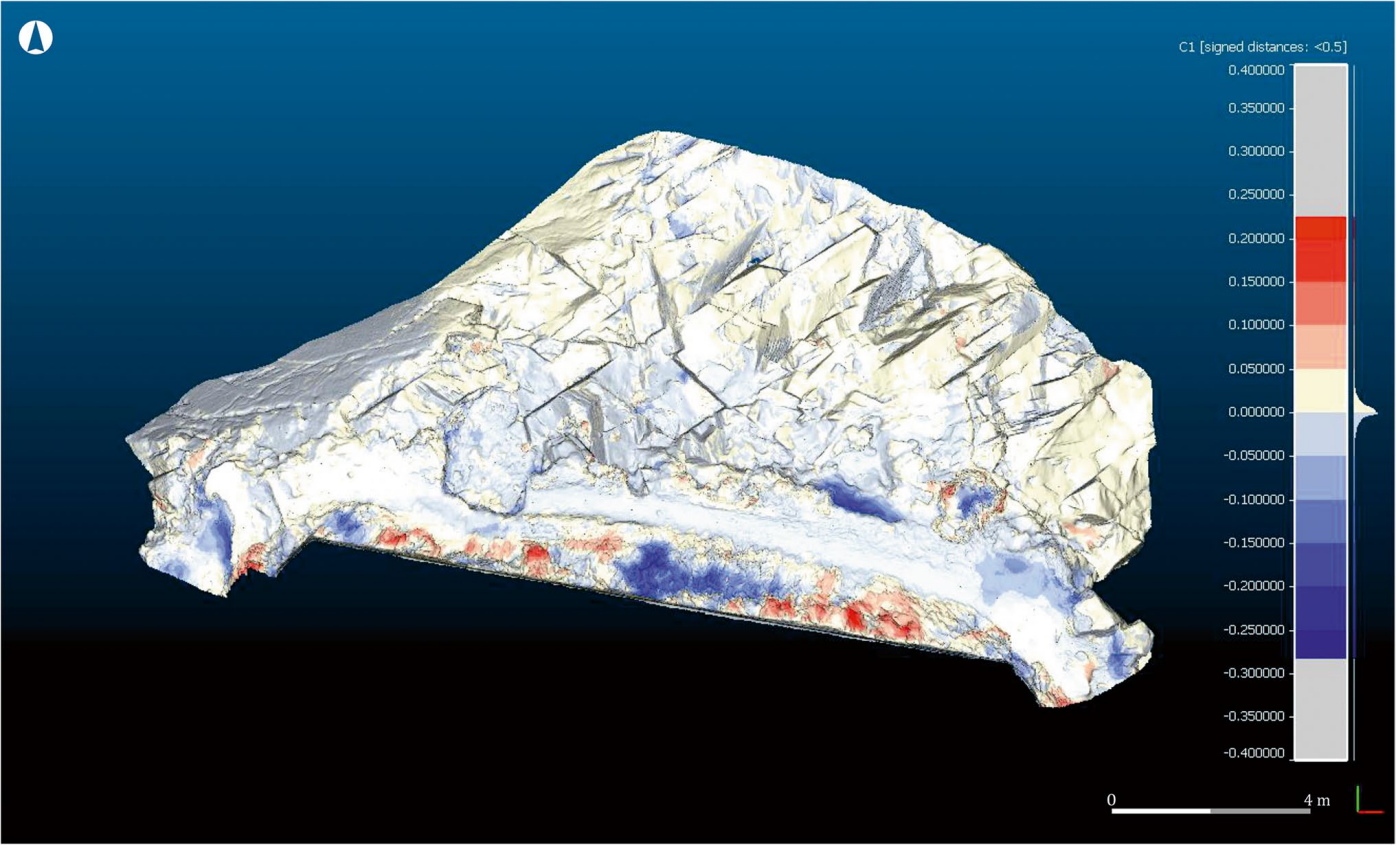
Model C1, where the loose (blue) and gain (red) of material can be seen, using as base for the representation the Sequence 2 (2017)

**Fig. 12:**
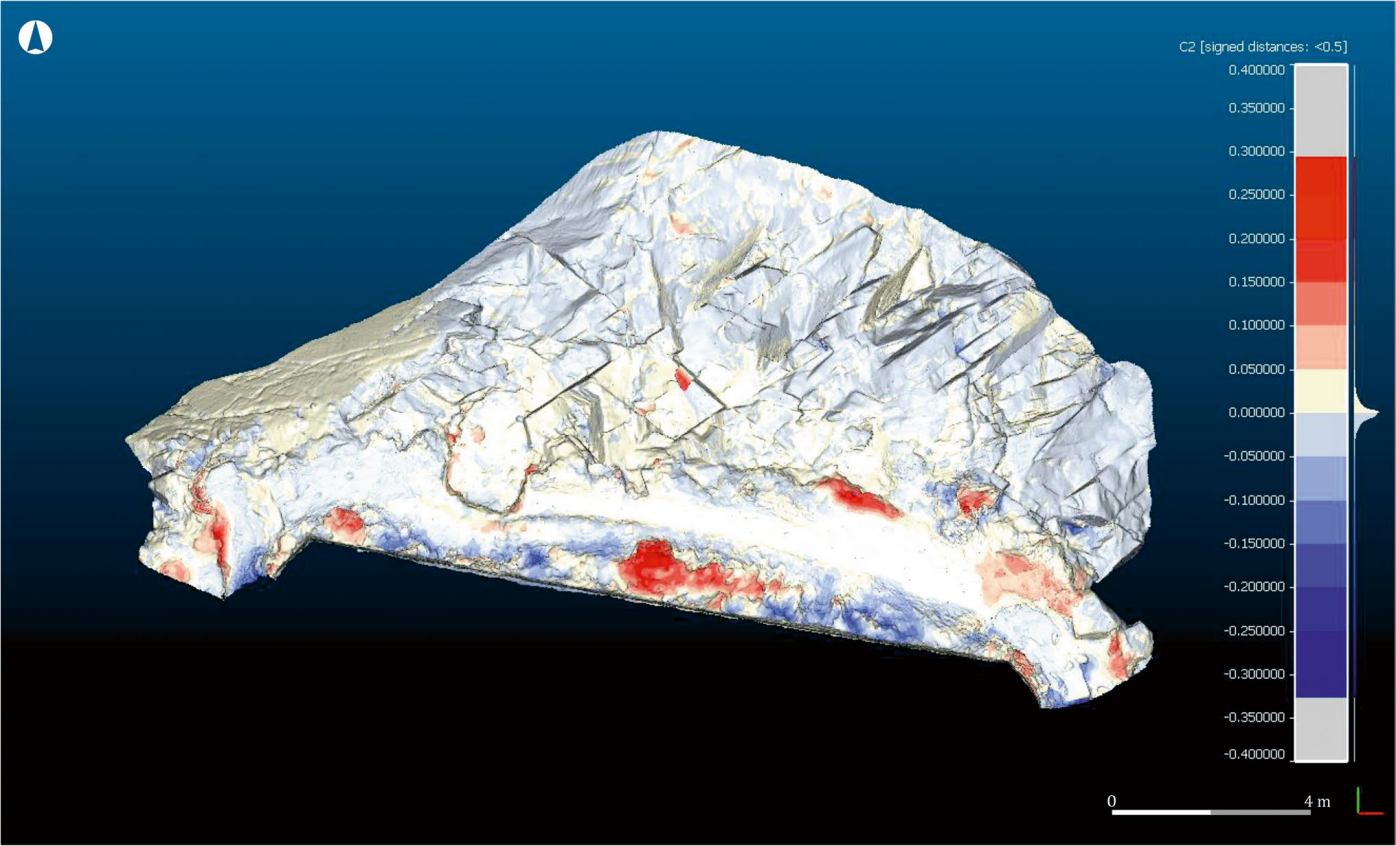
Model C2, where the loose (red) and gain (blue) of material along one year can be seen, using as base for the representation the Sequence 1 (2016)

The measure of the variations produced to the terrain by the erosion was carried out stablishing a regulated distance between both meshes of less than 0.5 m, as it was verified that none of the changes exceeded this measure. This way, CloudCompare© could quantify the addition of materials up to 50 cm and the loss of ground till minus 50 cm. The results were expressed by means of a colour scale, that depicts the differences measured in metres between the ground morphology of both models. The chromatic range of this scale was limited to red-yellow-blue, where red represents areas in which there has been an addition of material, and blue those in which there has been a loss of material. The intensity of the colour expresses the intensity of the associated process. This model was designated as C1 (Fig. 11). Using the same parameters of distance and chromatic scale, a second comparative model was generated, inverting the position of the sequences. As such, Sequence 2 became the base model, and Sequence 1 the comparison model. This allowed to identify some of the changes undergone by the site over the course of the year, not clearly visible in C1. This new comparison model was designated as C2 (Fig. 12).

It should be noted that to secure a correct measure of the differences between both models, the meshes were imported to CloudCompare© in real-life scale (1:1). Thereof, the comparison displays differences of centimetres on the ground morphology, with an accuracy or error range of ≈3 mm.

## Results

**Fig. 13:**
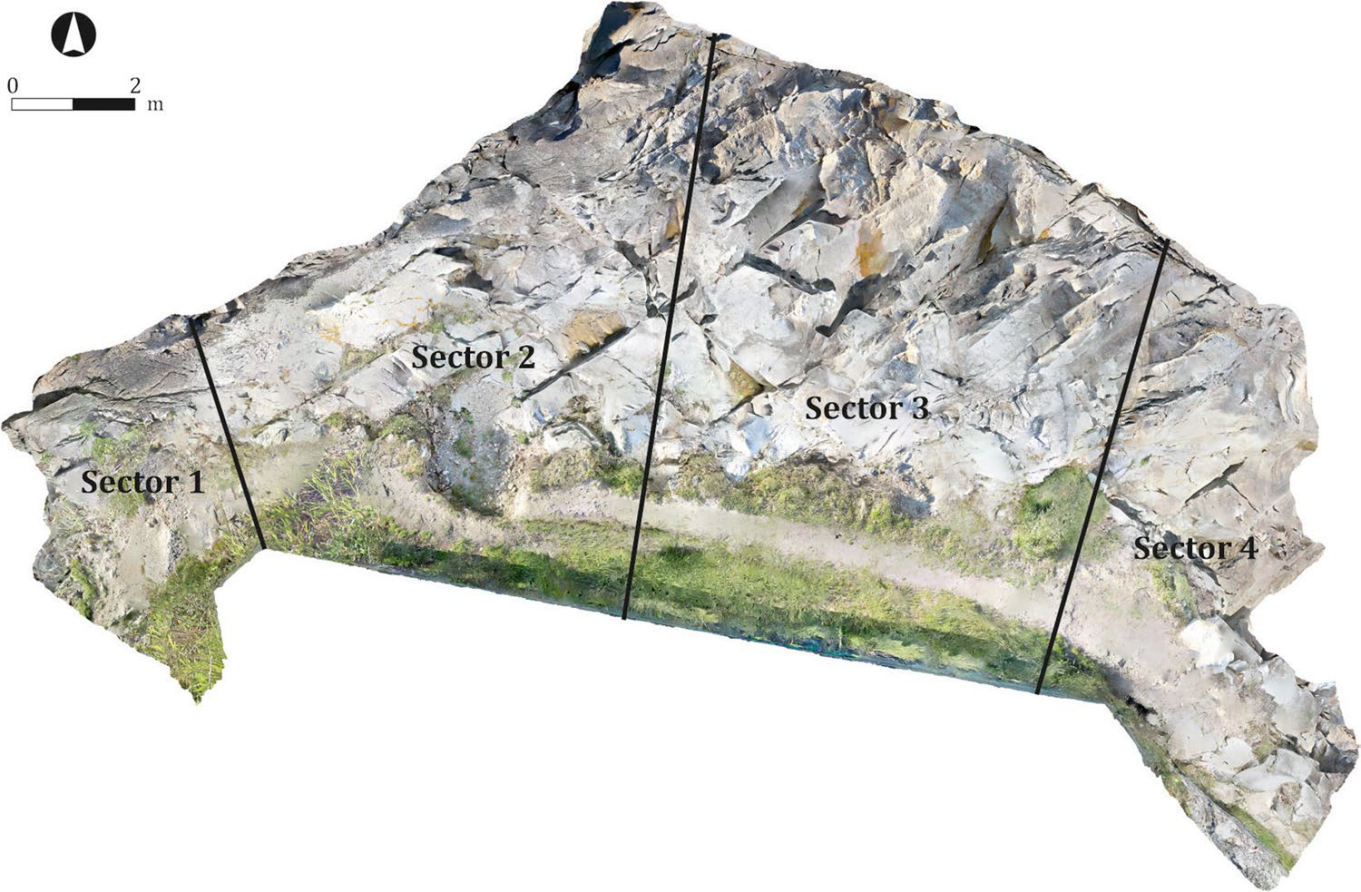
Compartmentalisation of the site for its detailed analysis

**Fig. 14:**
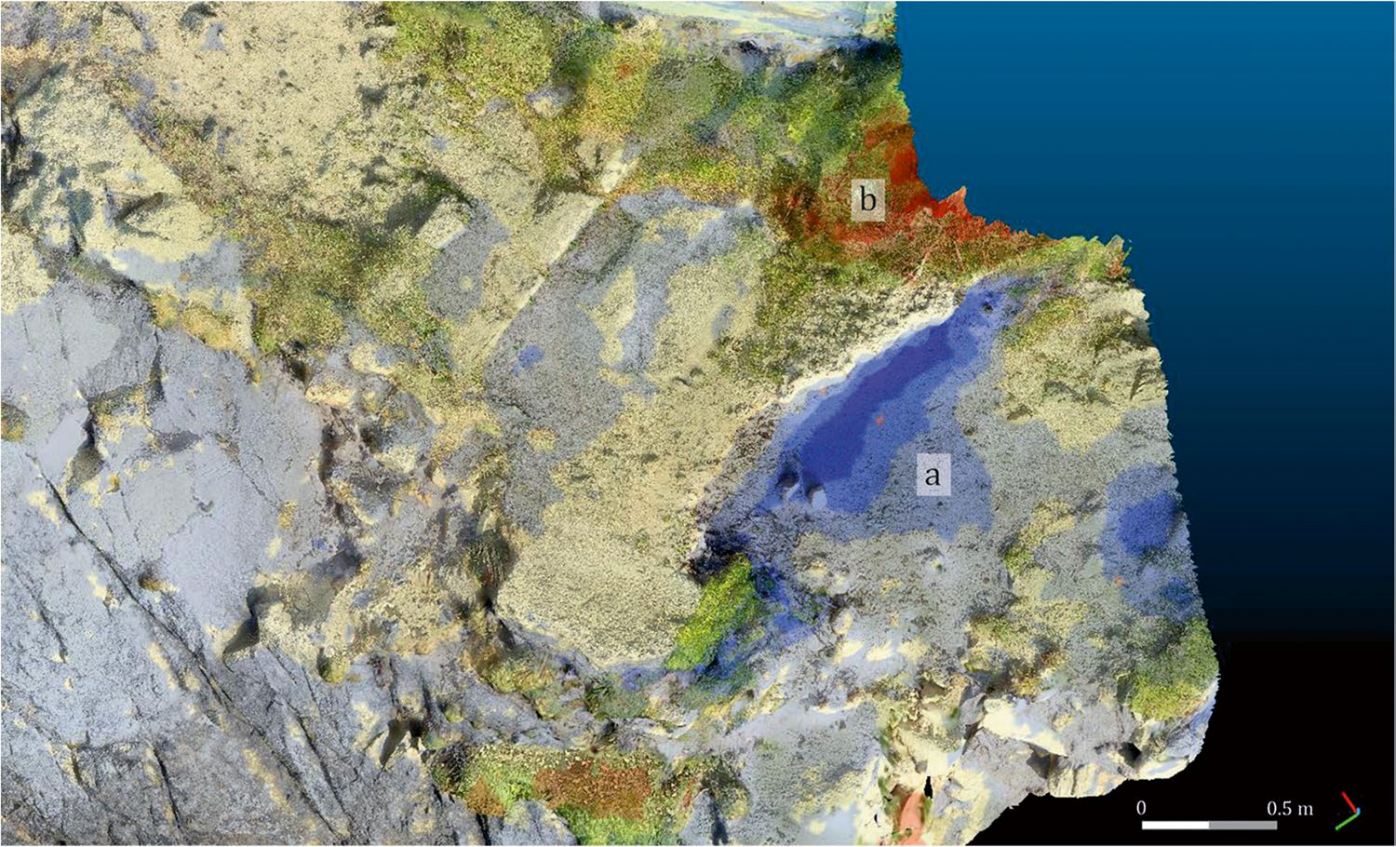
Detail of the changes produced in Sector 1, using as reference the model C1. Areas with loose (**a**) and gain (**b**) of material. Oblique view from the northwest

The results fully confirm the initial on-the-ground observations, while also highlighting other processes that are not visible to the naked eye. Between May 2016 and May 2017, the site was affected by marine erosion as well as by other human and natural factors. A slight loss of volume on the north-eastern face of the rocks is attested (light blue), as well as at other sectors of the site, especially in the western quartz pavement, some areas of the pathway and the layer of soil that still partially cover the rocky outcrop (dark blue). The site was divided into four sectors (1–4) for more in-depth analysis. From left to right (west to east), the following situations are attested (Fig. 13):

Sector 1: In this area, the Roman period fills that level out the north-western corner of the building and the quartz mortar belonging to a salting cistern have suffered severe losses (Fig. 14a). Total losses amount to 0.067 m^3^, with the mortar being especially affected: a loss of several centimetres in thickness was noted. The area is exposed to wind erosion and, occasionally, to the direct impact of the sea, which sometimes rises to this level. However, the most important factor for this sector is the transit of humans and animals between the beaches. The steep slope also exacerbates the effects of erosion, as the mortar is used as a step. A marked increased in the volume of vegetation is also attested (Fig. 14b).

**Fig. 15:**
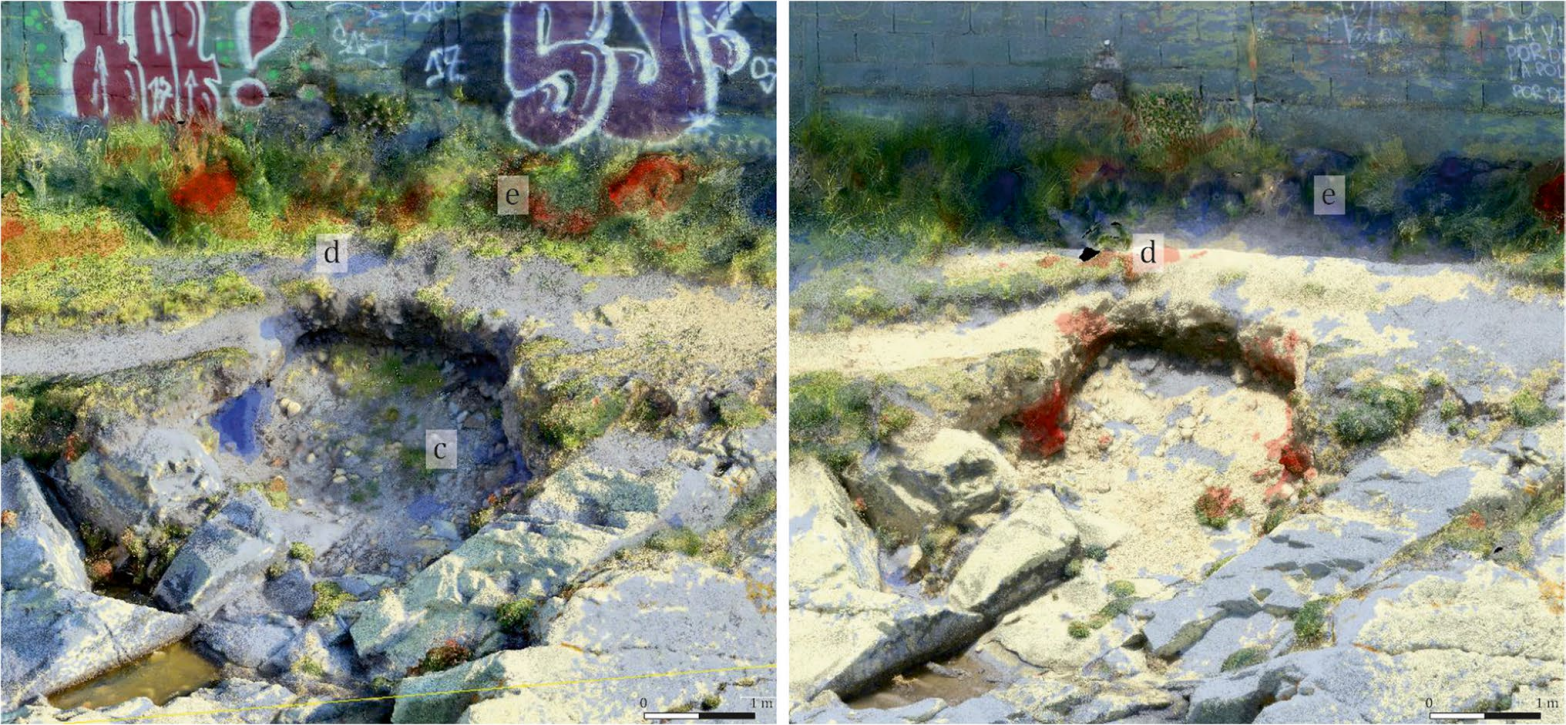
Detail of the changes produced in Sector 2, using as reference the model C1 (left) and C2 (right). Areas with loose of material (**c**) and (**d**), area with a gain of material (**e**). Oblique view from the northwest

Sector 2: In this area, the topsoil that sits over the rocky promontory, covering the site, has suffered extensive losses. Especially affected is the sinkhole located in the central area of the sector, as the comparison of both models clearly reveals (Fig. 15c). The losses are severe, amounting to approximately 0.35 m^3^ of soil. In addition, the area around the pathway has suffered moderate losses (Fig. 15d), owing to the decreasing width of the thoroughfare caused by the loss of material. The increase in vegetation in this sector (Fig. 15e) is remarkable, especially in the area surrounding the chalet’s walls.

**Fig. 16:**
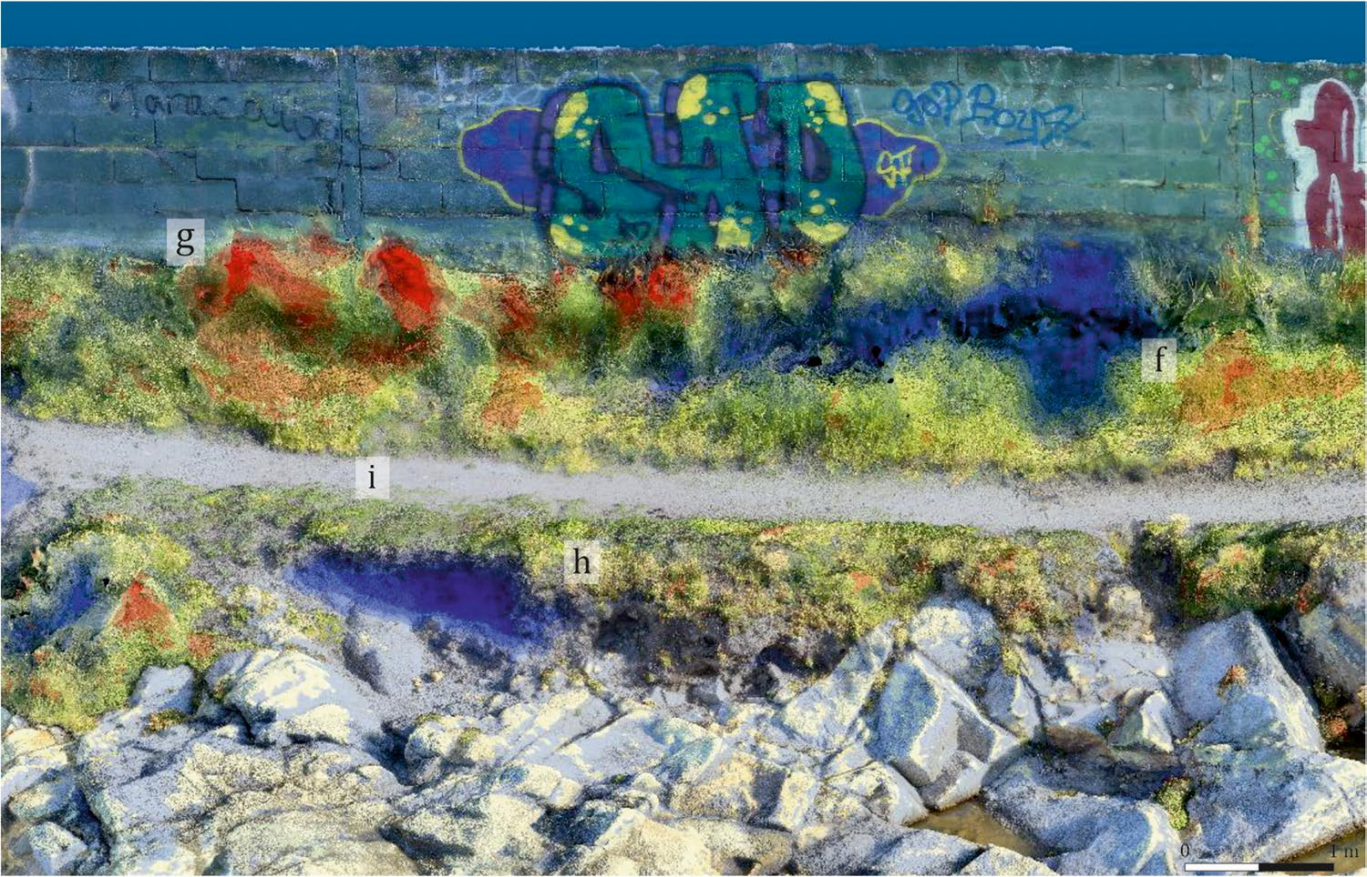
Detail of the changes produced in Sector 3, using as reference the model C1. Areas with a loose of material (**f**), (**h**) and (**i**); areas with a gain of material (**g**). Frontal view from the north

Sector 3: In this area, the sector attached to the chalet’s wall has suffered considerable losses (Fig. 16f), while vegetation has proliferated to a great extent (Fig. 16g). Important losses are also detected in the sea-facing front (Fig. 16h), and moderate losses in the pathway area (Fig. 16i).

**Fig. 17:**
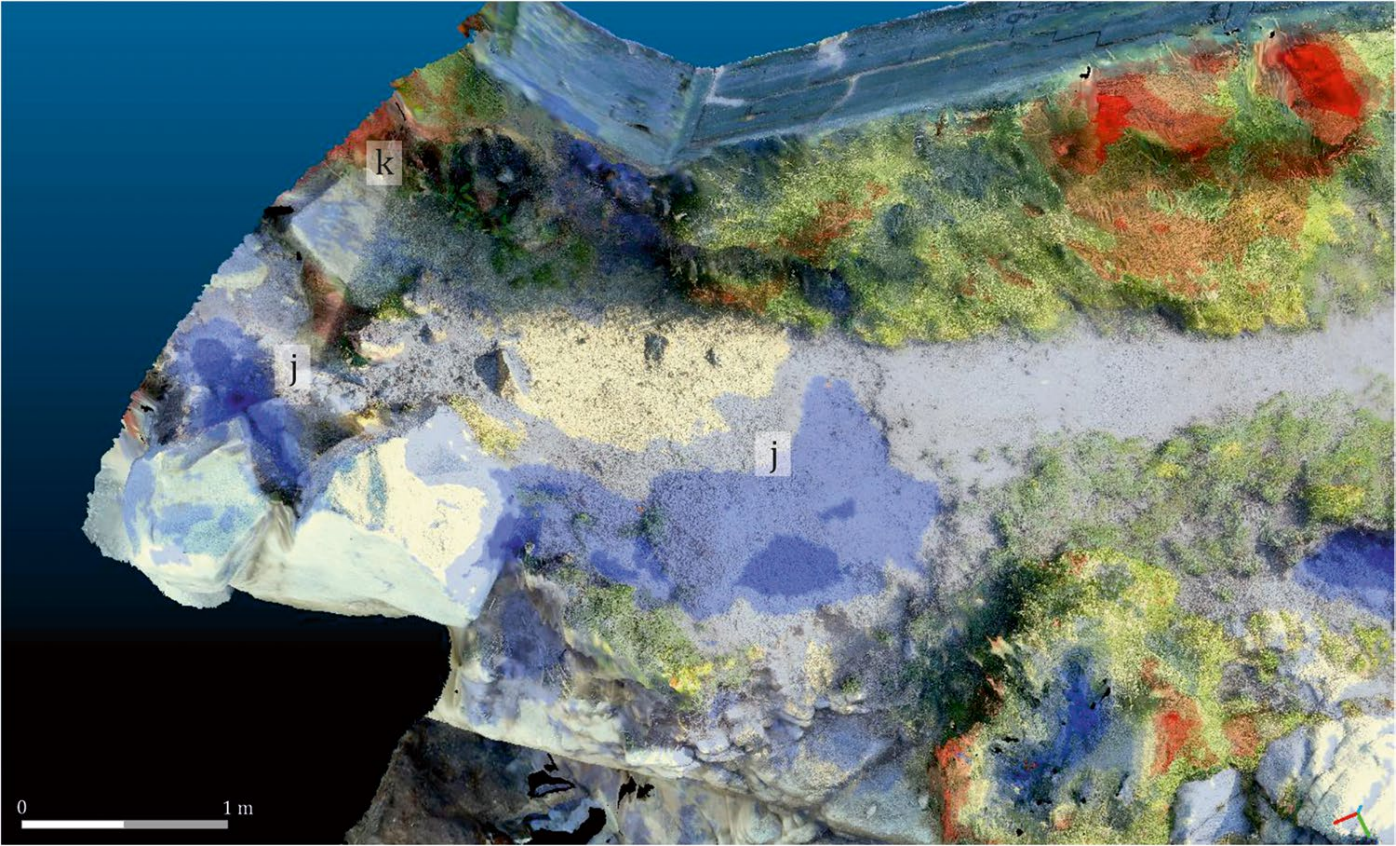
Detail of the changes produced in Sector 4, using as reference the model C1. Areas with a loose of material (**j**) and áreas with a gain of material (**k**). Oblique view from the northeast

Sector 4: Along with Sector 1, this is among the most severely affected areas of the site. Considerable losses are attested in the eastern corner and the whole eastern sector more broadly (Fig. 17j), while some areas present evidence of gain (Fig. 17k), probably owing to the displacement of eroded material from the blue areas. Afterwards, a draining channel running from the swimming pool, which operated as an added erosive agent, was detected. A considerable increase in vegetation is also attested.

**Fig. 18:**
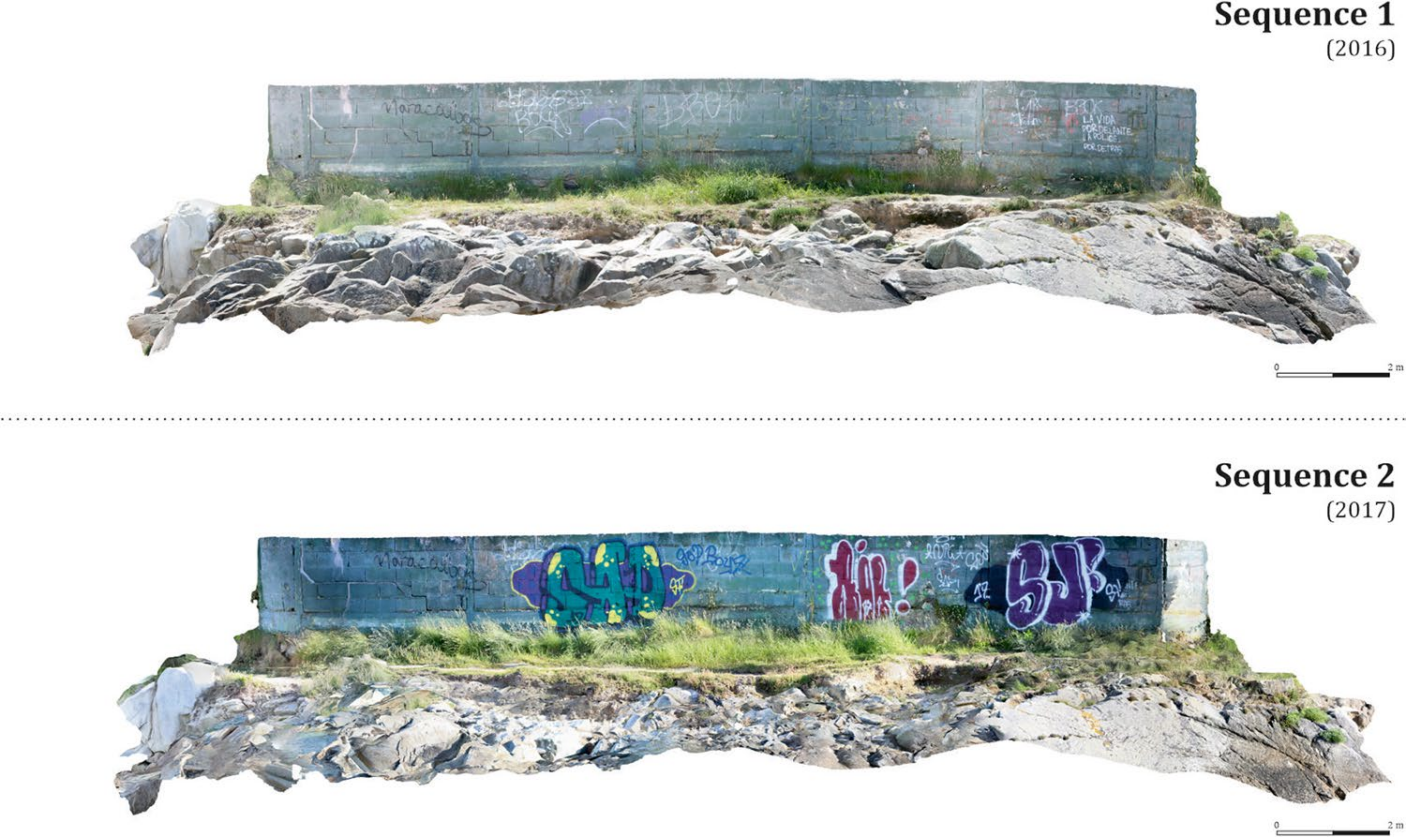
Changes and anthropic aggressions suffered by the site, visible after the processing of both photogrammetric models. Frontal view of the models, from the north

In general, a substantial increase in vegetation is detected over the whole site, especially around the perimeter wall that marks the boundaries of the private property, as well as other smaller areas scattered throughout the site. These are the areas where the sea has deposited new soil material (Fig. 10). Also clear is the generalised degradation of the most exposed areas of the rocky promontory (light blue). Finally, although this cannot be visualised using our methodology, the generation of the model also allowed us to detect an additional cause for concern: the ongoing graffitiing of the wall surrounding the private property, as reflected in the photographic sequences (Fig. 18).

## Mitigating measures: archaeological work and preservation of remains

The information obtained after generating and analysing the 3D models of Sobreira contributed to raising social and political awareness of the imminent danger to which the site was exposed. Based on the model, a series of mitigating measures to halt the deterioration of the factory were implemented. These measures had three main aims: to collect archaeological information, to address the agents responsible for the deterioration of the site, and, for the future, to monitor its evolution over time.

**Fig. 19:**
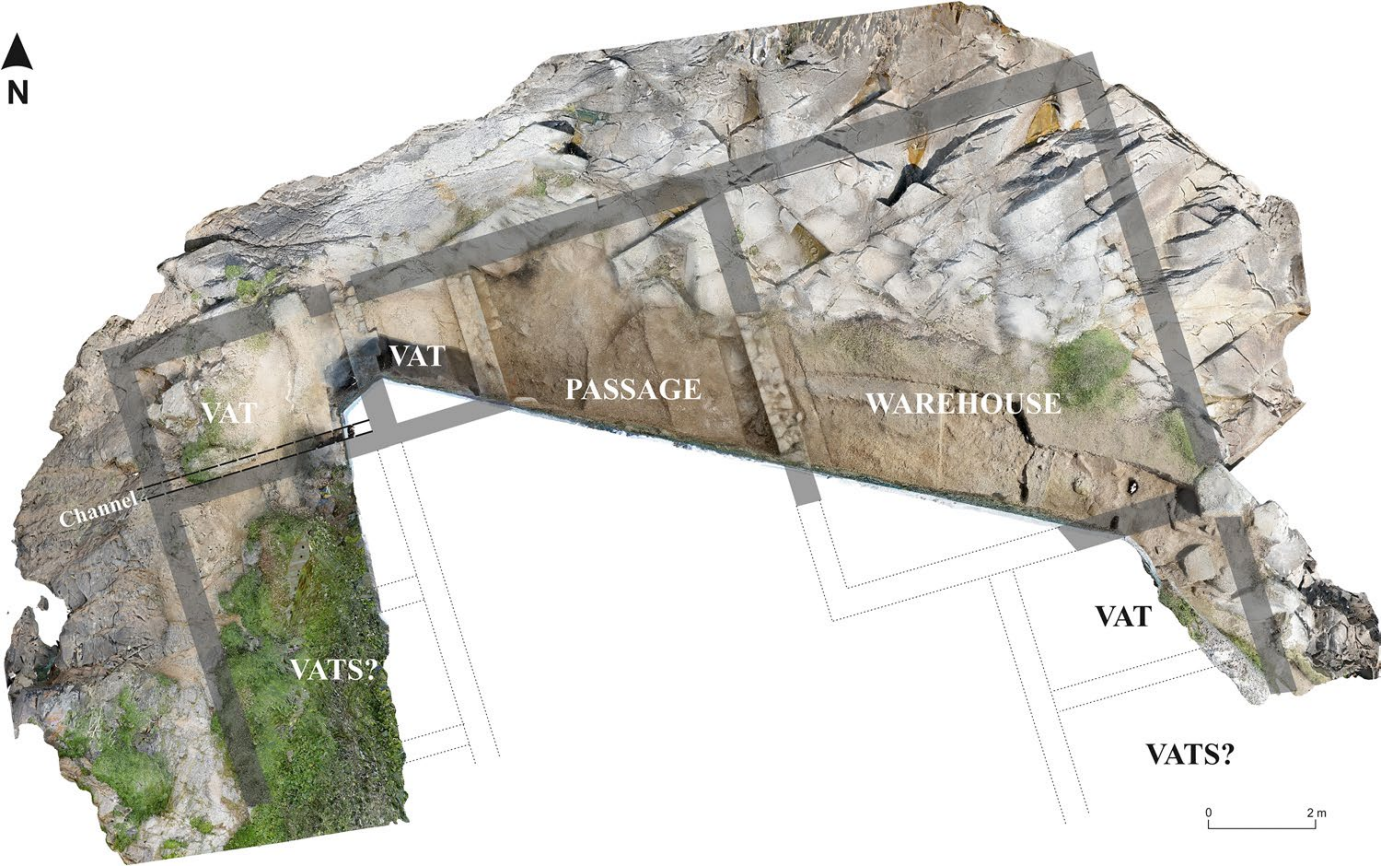
Plan of the factory

Given the severe threats to the fish-salting factory of Sobreira, it seemed necessary to undertake an archaeological excavation to record features and materials. This involved traditional archaeological recording methods and photogrammetry, which were used to produce detailed plans and a 3D model (Fig. 19), as well as a large number of photographs. This allowed for the recording of a large quantity of archaeological information that was in imminent danger of being lost as a result of the erosive processes affecting the site.

This work took approximately 2 weeks, one for excavation and one for conservation and restoration, and the results were important. From a scientific point of view, the excavation revealed unknown aspects of the site, such as the remains of several salting vats, which confirmed the interpretation of the site as a fish-salting factory, and not a *villa a mare*, as it had originally been interpreted (Hidalgo Cuñarro and Viñas Cué 1999: 88–89). The recording of the wall remains allowed us to reconstruct the complex’s northern face and estimate the size of the areas which are now lost, as well as to evaluate changes in coastal physiognomy (Fig. 19). In addition to the salting vats, the excavation identified other structures in the factory, including a draining channel connecting the inner areas to the sea, which was probably used to dump the factory’s waste into the ocean (Fig. 19). It is estimated that approximately 73 m^2^ of the Roman building hare now lost, probably as a result of marine erosion, and this has changed the physiognomy of the rocky promontory between the beaches since several metres of it have been lost, from the Roman period to today (Fig. 19). It must be taken into account that, at certain times during the winter, in adverse weather conditions—storms, episodes of explosive cyclogenesis, etc.—the waves beat the modern wall built over the Roman remains. Some of the northern sector, the sector most gravely affected by these agents, is entirely covered by the sea during high tide. Unfortunately, we do not have detailed records of the past condition of the site—before 2016—and so it is difficult to estimate whether the deterioration of the site has taken place in recent times—due to climatic changes—or whether the loss of the north-eastern corner happened centuries ago.

**Fig. 20:**
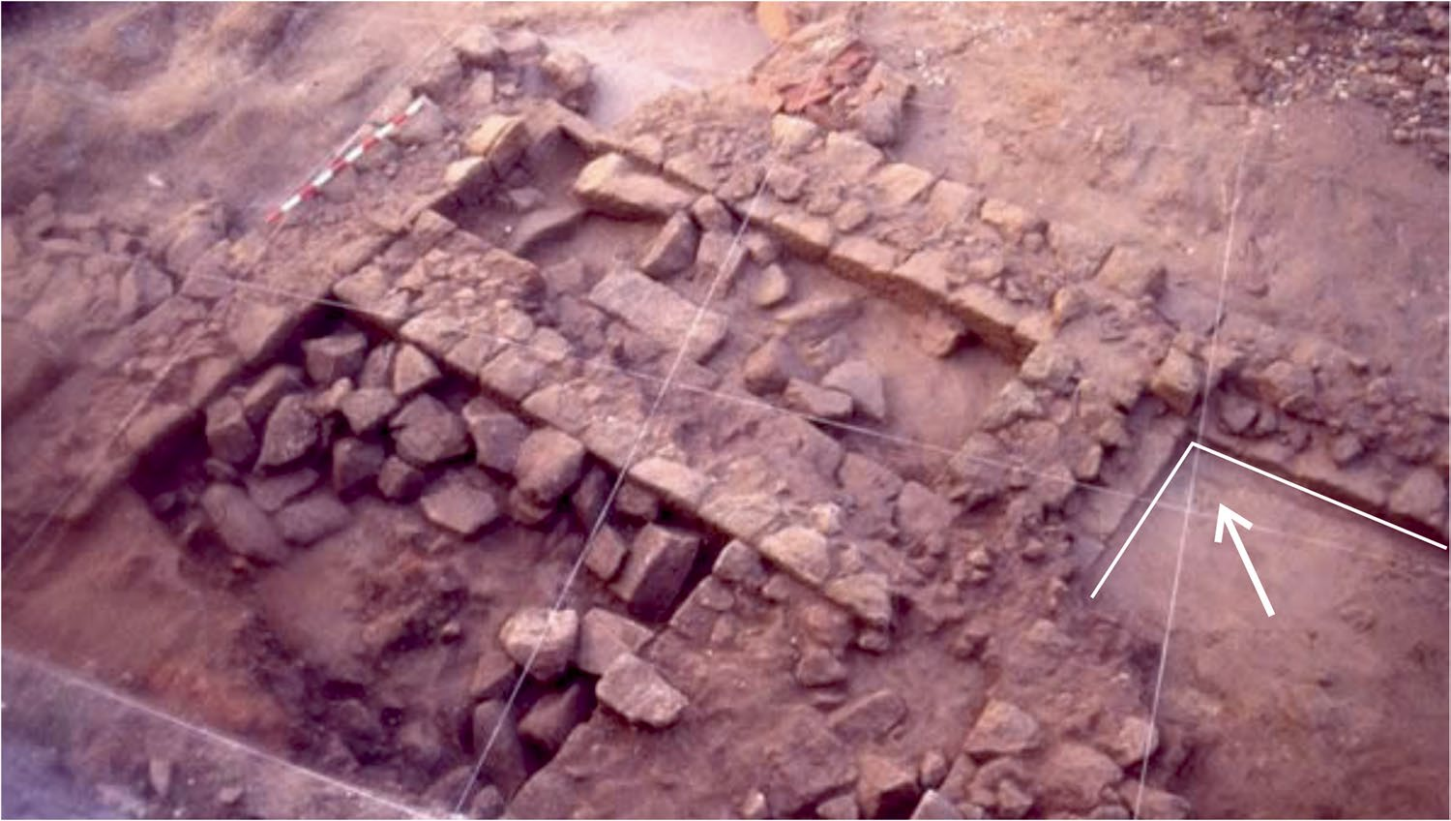
Reinforcement in the foundations of the walls in some fish-salting factories: O Cocho (Author: J.M. Hidalgo Cuñarro; Archive: Quiñones de Leon Museum)

**Fig. 21:**
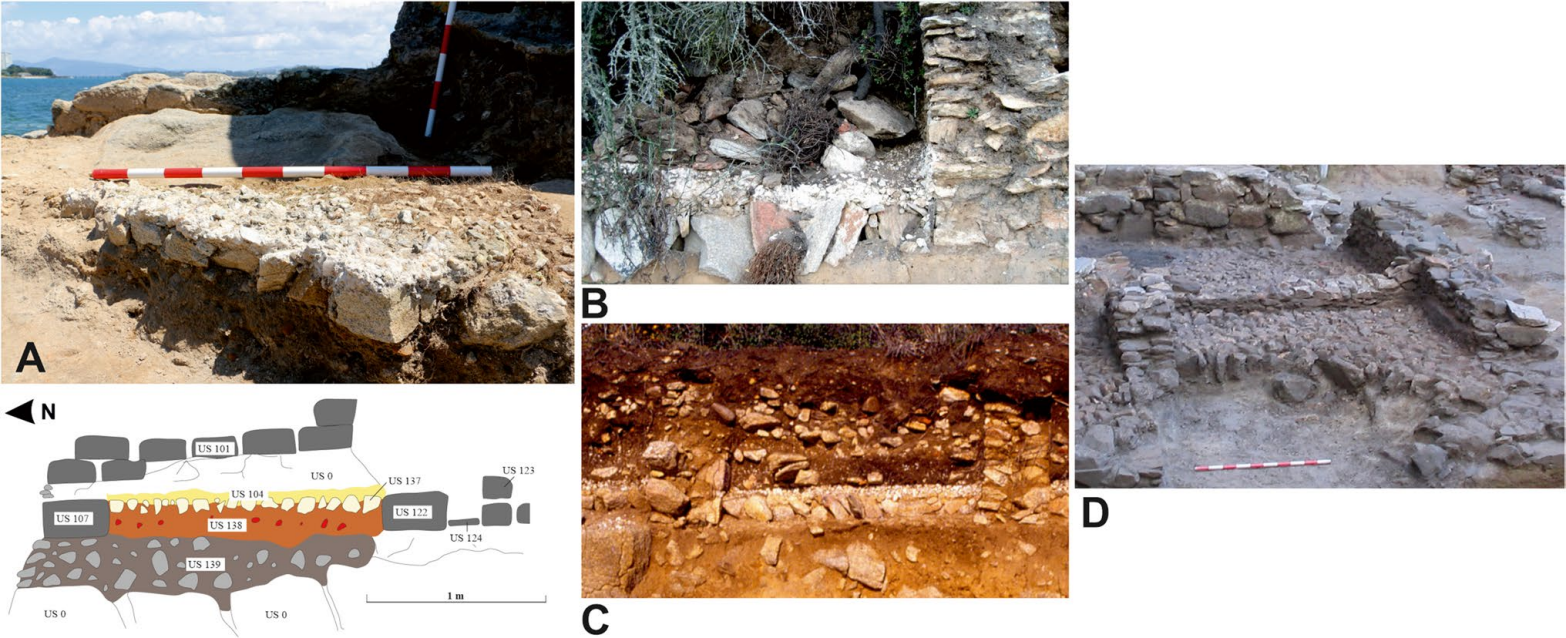
Example of the construction of a vat. (**A**) Photo and stratigraphic cut of one of the vats from Sobreira; (**B**) a vat of the factory of Canexol (Ons); (**C**) stratigraphic cut of a vat of Cariño; (Cariño) (**D**) foundation with rammed stones inside a vat of Praza de Compostela (Vigo)

The excavation also revealed the depth of the structures and important parts of the complex, such as the aforementioned channel between the passage and the sea, as well as the presence of reinforcing walls near the vats—a type of feature also observed by us in other Galician fish-salting factories as Fiunchal, Marqués de Valladares or O Cocho (Fig. 20). The constructive system of Sobreira’s vats can be found also in other factories, as O Canexol, Praza de Compostela or Cariño (Fig. 21). Once the walls were constructed, an inside foundation is created through granite blocks rammed in a diagonal axis, covering them with a limestone and quartz mortar. Finally, the walls are covered with a thin limestone mortar, completely lost in Sobreira, but still visible in other factories as Adro Vello.

**Fig. 22:**
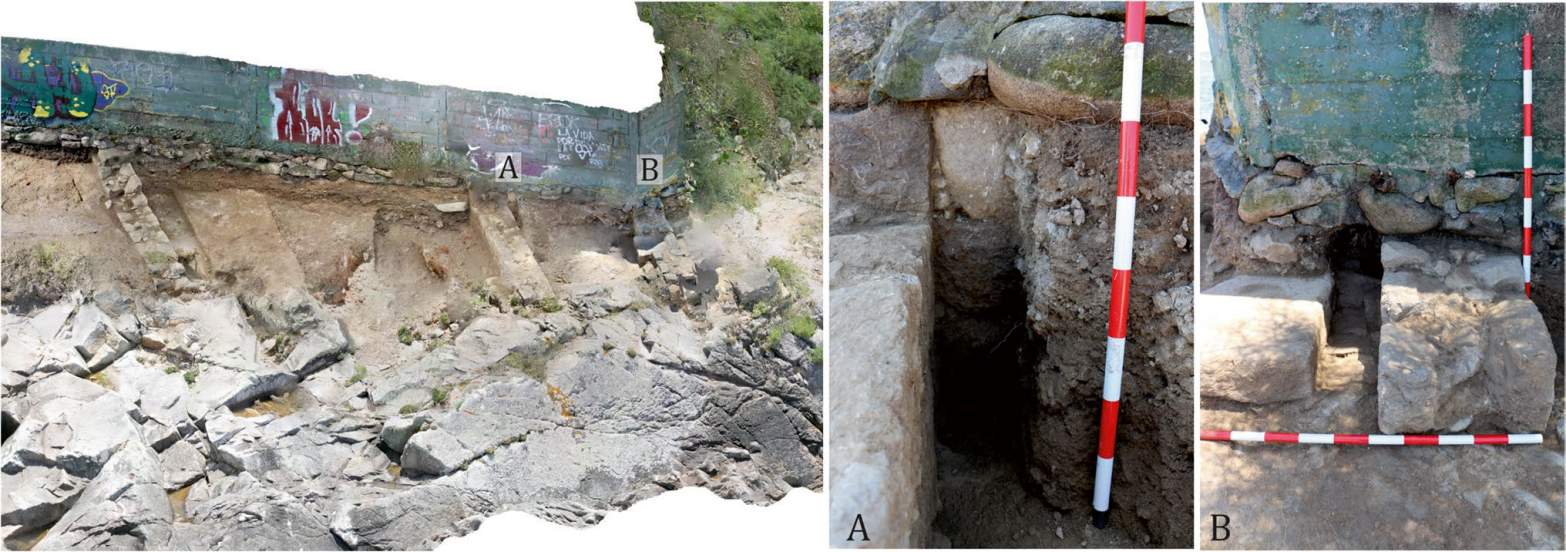
Detail of the excavated area, where it is visible how the roman walls run under the enclosure of concrete slab of the swimming pool

The final plan of the factory shows probably only a small part of the building, being the north part destroyed by the action of the sea and the southern by the construction of a chalet’s swimming pool and a tennis court (Fig. 22). However, this small part of the building suggests that it could be a factory with a “U” shape or with a longitudinal plan. If Sobreira had a “U” shape, a common factory model in the Rias Baixas, visible for example in Bueu (Díaz García 2015; Fernández Fernández and Díaz García 2017), the vats would be located to the east and the west, with an empty central area, used as a courtyard. However, the disposition of the walls and the vats (Fig. 19) indicate that the factory had a rectangular or longitudinal plan located over the rocky outcrop, with a battery of vats on both long sides—east and west—that would finish in the western corner, attending to the presence of a small vat in the northern façade, acquiring, therefore, an “L” shape. A corridor would allow access to the vats and the north-eastern corner could be occupied by a warehouse (Fig. 19).

The disposition of the building is common among the factory patterns detected in *Gallaecia*. Small and aisled factories constructed over rocky outcrops in small beaches. The beach would work as a natural harbour, where the fish could be landed and transferred to the factories for the processing. Besides, in all the cases detected in Galicia, these factories were also located near a freshwater stream, that would be used to clean the fish. In the case of Sobreira, a spring or freshwater stream, located a few metres away in the beach of Fouciños, was probably used by the factory.

The material collected during fieldwork dates the activity of the site to between the second and early fifth centuries AD. Very scarce materials were recovered during the excavation, adding to their study others from a private collection, recovered from the site along the years, allowing to propose an accurate date of use. As the main dating elements:

- Various fragments of amphorae type San Martiño de Bueu 2, dated among the third and the beginning of the fifth century A.D. (Fernández Fernández 2017a)
- Painted wares, plain wares and a glazed mortar from Bracara Augusta and common in the context of the fourth century in the nearby *villa* of Toralla (Fernández Fernández 2014)
- Some fragments of ARS D1, among them a Hayes 61 individual, probably dated between the end of the fourth and the beginning of the fifth century (Hayes 1972).
- A micaceous cooking pot with a reinforced edge, handcrafted, dated on the first half of the fifth century (Fernández Fernández and Bartolomé Abraira 2016)
- More ancient materials, as some fragments of Early Imperial Hispanic Terra Sigillata, dated at the beginning of the second century.
- And Roman glass (Isings 106 and 126) dated between the third and the beginning of the fifth century (Isings 1957)

The chronology of Sobreira matches with the data about other nearby fish-salting factories. For example, the contexts of the factory of Marqués de Valladares state that it was constructed during the first half of the second century and abandoned during the first third of the fifth century (Fernández Fernández 2014: 34). A similar chronology presents Bueu (Díaz García 2015), while Adro Vello is probably constructed at the end of the first century A.D. and Igresiña is abandoned at the end of the fourth century (Gorgoso López and Acuña Piñeiro 2016). Therefore, the chronology of use matches with the data provided by other factories, constructed at the end of the first century or more probably during the first half of the second century; and an abandonment at the end of the fourth or the beginning of the fifth century, coinciding with the general crisis of this kind of industrial establishments in the Atlantic (Bernal Casasola 2008).

**Fig. 23:**
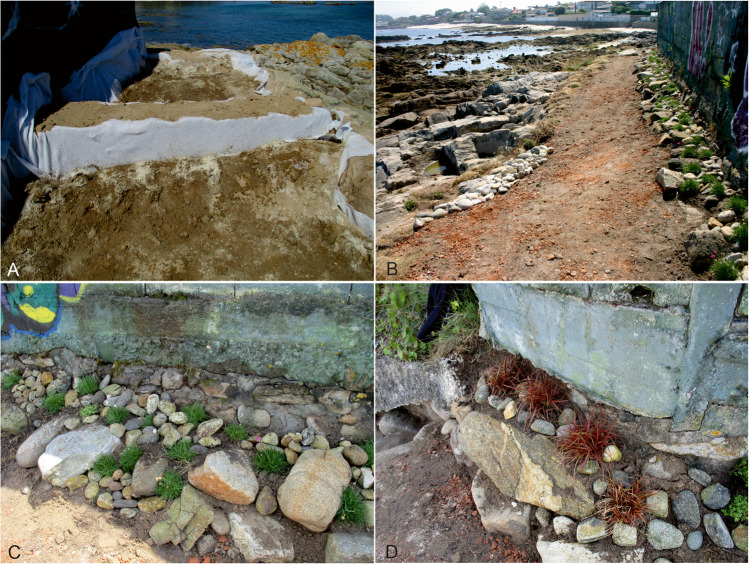
**A** Construction of protective structures. (**B**) Final result after the conservation-restoration process. (**C** and **D**) Detail of the species *Festuca glauca* and *Uncinia rubra* used to stabilise the terrain

After the excavation, measures were adopted to address the deterioration processes and give the site as much protection as possible. Protecting the structures from marine erosion was urgent: although erosion cannot be completely halted, the construction of two small walls with stones collected on site reduces its impact upon the standing structures. Furthermore, it was necessary to undertake measures to reduce human impact, which was very intense because of the path that links the beaches. Since it was impossible to stop people from using it, the path was covered and repaved, raising it by about 30–40 cm with soil from the site, bricks—from the Roman villa of Toralla—and limestone, in the hope that this will progressively stabilise the terrain and protect the structures. Also, in order to consolidate the new path and the surrounding terrain, the edges of the path were planted with undergrowth. Currently, at both ends, the path is accessed through newly built stone and mortar steps specifically laid down to protect the quartz mortar in the western corner (Fig. 23).

The construction of a private building had already destroyed part of the site, and so it was necessary to raise awareness, especially among local residents, about the existence of a Roman fish-salting factory in the area, and emphasise the fact that the site was rapidly disappearing. The intervention at the site, promoted by Vigo’s city council, and its publication in the local media, had a very positive effect in this regard, as most of the city’s inhabitants did not previously know of the site’s existence or of its preservation problems.

A visit to the site in March 2018, a year after works came to an end, revealed that, despite the work of the conservators, human action, and especially marine erosion, has continued to damage the site in the same areas in which their action had been attested previously. These factors, which are to a large extent inevitable, make it likely that the site will disappear in the near future. The measures that would be necessary to fully prevent this, for instance the construction of a concrete dyke, would have an enormous visual and environmental impact. It is for this reason that monitoring the site and the archaeological works undertaken as a result is so important: by monitoring carefully, we have been able to collect crucial information about the site, and the preservation measures have added a few more years to the life of this exceptional site. As the adoption of effective measures for the long-term preservation of the site is unfeasible, the 3D models will at least preserve a semblance of the site’s original morphology, and the data collected will be useful in monitoring similar processes in comparable sites, in informing mitigating measures and thus preserving the structural integrity of the sites as much as possible.

## Discussion

The monitoring of the erosive action of the sea was not one of the main proposes of the GaltFish project, as it was already stated before. However, it has been of great interest and aid to the project, providing a suitable example of the implications that the marine erosion and human action can have over the coastal archaeological sites.

Due to the results obtained by the comparison of the first two models or sequences, an archaeological intervention was needed to preserve the remaining information that the site could provide, before its definite loss. On the other hand, this circumstance refrained the realisation of a third model, that could have added even more information to the crescent erosion of the site, allowing the cross-reference of the outcomes with other data, as climatic variances. Despite this, the preservation of the site was considered a priority, leading to an archaeological excavation and preservation works that fairly changed the morphology of the terrain, not allowing the creation of this third model.

It must be stressed that a similar approach was adopted previously in both natural- and historical–heritage-related settings: for example, the monitoring of erosion on cliffs and rock walls (Abellán et al. 2011; Lim et al. 2005; Obanawa and Hayakawa 2018) and at underwater archaeological sites (Ferentinos et al. 2020; McCarthy et al. 2019), and the structural pathologies suffered by certain archaeological and architectural features (Benavides López et al. 2020; Lai et al. 2019; Tapete et al. 2013). Other works have similar aims, although they emphasise the theoretical and experimental dimensions (Daire et al. 2012; López-Romero et al. 2014) and use different tools and analytical approaches. In most cases, the chosen tool is a laser scanner, which is more costly, making the tool less readily accessible. The methodology proposed by Project GaltFish is based on tools which are both accessible and user-friendly, while yielding equally valid results, in this case with the aim of informing conservation strategies.

Taking into account the overall results, 3D photogrammetry is an efficient and functional tool for monitoring changes in archaeological sites over time. The tasks involved—taking photographs, generating the models and creating the compared model—can be carried out with standard archaeological equipment—cameras and computers—which makes it an easily accessible tool. The comparison of the models, which was undertaken with open-access software and comprising a simple procedure that consists of overlapping the models, can yield significant information on the effects of erosion and also permit its quantification over time.

Although the marine erosion of the Galician coast is nowadays really aggressive, the time lapse between both models brings enough results to allow a first approach to the site. Monitoring how the climatic changes of the four seasons affect the site, rather than only the winter phenomena, the more violent ones (spring tides, cyclogenesis, rainfall increase or strong winds). As stated, the human pressure and even the vegetation growth are erosion factors to have in consideration, occurring more likely during the spring and summer months. Hence, the space of a year between the first comparison models seems to be a suitable lapse of time for this type of site, adjusting the successive sequences to the results obtained through this first comparison.

The simplicity of the method and the use of common tools allow to easily reproduce the procedure, opening also the possibility to combine it with other monitoring methods or even its application over other non-coastal archaeological sites or even over natural heritage. The time lapses can also be variated, being possible to do as many models as needed to monitor the changes suffered by the terrain or the remains, as well as the accuracy of the surveys, subjected to the needs of each case study. An easy and affordable tool that can provide notable information to the researchers, as it was already demonstrated along this paper with the case of Sobreira.

The archaeological excavation allowed definitely to characterise the site as a fish-salting plant, joining the nearby and already known examples of Fiunchal and O Cocho, as well as the ones located in Vigo’s inner city—Marqués de Valladares and Praza de Compostela—and the factory of Igrexiña, in the opposite side of the Ría de Vigo. This fish-salting plant would develop simultaneously to another huge industry: the production of salt though solar evaporation. Arguably, the Ria of Vigo gathers the biggest known group of Roman salt pans. Aside from the large salt complex of O Areal (Castro Carrera et al. 2019; Iglesias Darriba et al. 2017), similar remains were discovered in Punta Toralla (Pérez Losada et al. 2008: 496) and in Bouzas inlet. There is no doubt about the relation between the salt and fish-salting complexes, since both types of factories are in use from the first to the third–fourth century A.D., with the salt factories supplying the necessities of the fish-salting complexes. These industries are part of the industrial coastline of Vigo during the Roman period, along with *villae a mare*, *agglomérations secondaires*—as the centre of Vigo-*Vicus—*and hillforts, specialised in fishing activities and in the production of salt and fish-salting products, as well as harbouring and goods redistribution functions.

During this time, a significant number of the population would still live in villages located on high grounds (Castro de Vigo, A Guía, Candeán, Toralla, Alcabre, Castriño…). The seaside and anchorages would progressively be used not only as trade harbours, but also as new living and production areas, with the installation of the fish-salting factories and the largest preserved salt production complex of Roman time, resulting in a vicus with trade and industrial functions (Fernández Fernández 2016a, b: 25). The identification of Sobreira as a fish-salting factory adds weight to this idea, as it is located further south than the factory of O Cocho and the salt complex of Toralla, expanding the industrial coastline and demonstrating that the Ría of Vigo was, during the Early Roman Empire, one of the most active harbours in the Atlantic façade (Fernández Fernández 2014) and one of the main salt and fish-salting production centres.

Sobreiras’ chronology reinforces likewise the hypothesis of a generalised abandonment of the salt-fish productions in the Northwest between the end of the fourth and the beginning of the fifth century. The cease of the salt production would have occurred earlier (Iglesias Darriba 2009: 229), probably due to an adverse climatic change—rising sea levels and a progressive increase of rainfalls—happened during the third century and also observed in the northern coast of Portugal (Granja 2013). The end of the salt factories does not imply the end of the fish-salting production but had probably weakened the industry, as it was necessary to import salt from other coastal sites. However, not long after the fish-salting factories of the Rias Baixas would cease also their production, perhaps because of a generalised crisis, being progressively abandoned, as happened as well with Sobreira.

## Conclusions

The monitoring of the archaeological site of Sobreira (Vigo) through the generation and comparison of two 3D models separated by a year has proven to be an efficient tool for assessing erosive processes affecting the site. Once these processes had been assessed, we were then able to design measures aimed at palliating the effects of erosion. The methodology followed has great potential for the management of this sort of coastal archaeological site, which is exposed to a wide array of erosive processes. The tool can be used by public agencies for the analysis and protection of coastal heritage.

Our results preceded and informed the excavation of the site, which was itself followed by the implementation of conservative preservation strategies, since fully protecting the site against marine erosion was deemed unfeasible (for economic reasons and for the visual disruption that such an undertaking would cause). Our work also aimed to raise awareness about the presence of the site and its relevance by ensuring that the local media published stories on the site, the archaeological work being carried out and the measures being adopted to protect it. This contributed to the frequent users of the pathway, who were a factor of erosion, being more aware of the nature of their surroundings, and many decided to start bypassing the site, reducing the degree of human impact.

The recovery of the lost archaeological data can be added as a result, as well as to the social awareness and the increase of the scientific knowledge of the Roman past of the Ría de Vigo and this type of industrial facilities. Overall, the proposed tool seems like an effective method to monitor menaced archaeological heritage, a low-cost and easy method, with accurate results and more accessible than other tools.

Furthermore, the 3D models allow to preserve in digital format the monitored sites, creating photogrammetric material that can be incorporated into the heritage management lists, so they can be used with scientific, informative and administering purposes. The comparison of the models reveals an image of the past (analysing the erosion agents), of the present (monitoring and current state) and also of the future (decision-making processes over the different protection measures) of Sobreira.

**Fig. 24:**
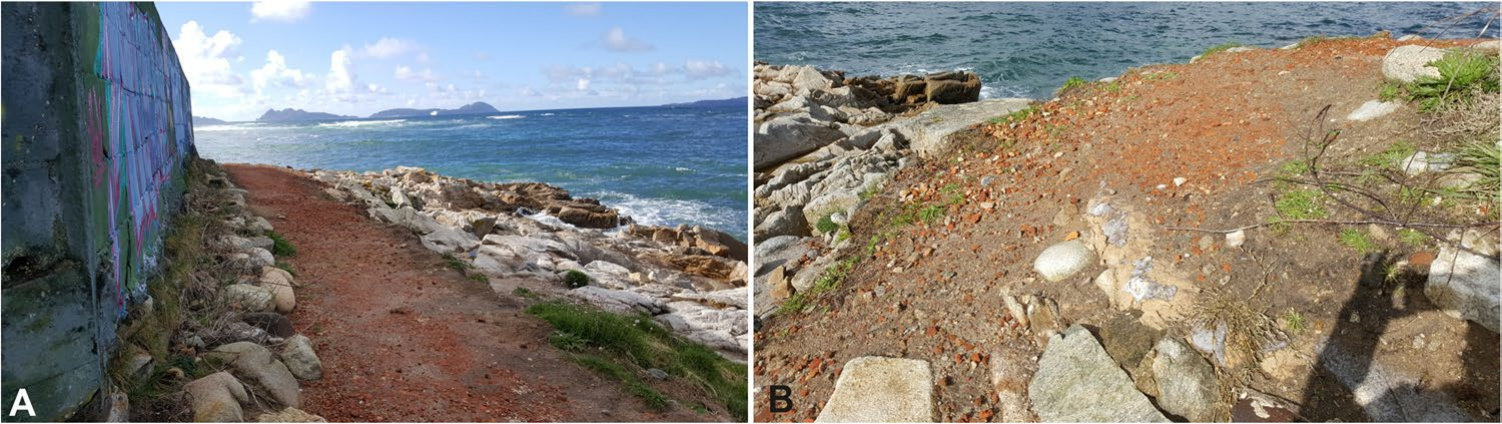
**A** View from the east of the intervention area, taken in March of 2018. **B** Detail of the mortar area, where the loose of material can be seen

The visit to the site, a year after the finalisation of the works, demonstrated the positive effects of the measures undertaken (Fig. 24). In addition to collecting important historical and archaeological data, the project has contributed to palliating the effects of erosion on the structures. Although the effects of erosion continue (loss of soil and of structural volume), the situation is much improved and the site will survive for years, unless weather conditions take a violent turn for the worse.

## Availability of data material

Non applicable.

## Data Availability

Non applicable.

## Footnotes

1 O Cocho, Fiunchal, Praza de Compostela and Marqués de Valladares in Vigo; Igrexiña (Nerga); A Pescadoira (Bueu); O Canexol (Ons); A Lanzada (Sanxenxo); Adro Vello (O Grove); Alobre (Vilagarcía de Arousa); Aldea de Cariño (Cariño); Punta dos Prados (Espasante); Porto de Bares (Bares); Praia de Area (Viveiro) and Plaza del Marqués (Gijón).

2 Like the sites of Vilar (Vigo), Panxón (Nigrán), Noville (Mugardos) or Centroña (Pontedeume) (Lomba Portela 1987; Fernández Ochoa and Martínez Maganto 1994; Suárez Piñeiro 2003).

3 Like the sites of Angeiras in Matosinhos (Almeida 1969) and Alto de Martin Vaz in Povoa de Varzim (Fortes 1969), traditionally considered Roman fish-salting factories, but that could be considered dubious given the information gathered.
